# Self-assembled DNA nanostructure containing oncogenic miRNA-mediated cell proliferation by downregulation of FOXO1 expression

**DOI:** 10.1186/s12885-022-10423-8

**Published:** 2022-12-20

**Authors:** Avishek Kar, Kanchan Kumari, Sandip K. Mishra, Umakanta Subudhi

**Affiliations:** 1 DNA Nanotechnology and Application Laboratory, CSIR-Institute of Minerals and Materials Technology, 751013 Bhubaneswar, India; 2 Academy of Scientific and Innovative Research (AcSIR), Uttar Pradesh 201002 Ghaziabad, India; 3 Department of Molecular Biology, Umea University, Umea, Sweden; 4 Cancer Biology Laboratory, Institute of Life Sciences, 751023 Bhubaneswar, India

**Keywords:** DNA nanostructure, FOXO1, miRNAs, Breast cancer, MCF7 cell line, P21, P27

## Abstract

FOXO1 transcription factor not only limits the cell cycle progression but also promotes cell death as a tumor suppressor protein. Though the expression of FOXO1 is largely examined in breast cancer, the regulation of FOXO1 by miRNA is yet to be explored. In the current study, self-assembled branched DNA (bDNA) nanostructures containing oncogenic miRNAs were designed and transfected to the MCF7 cell line to decipher the FOXO1 expression. bDNA containing oncogenic miRNAs 27a, 96, and 182 synergistically downregulate the expression of FOXO1 in MCF7 cells. The down-regulation is evident both in mRNA and protein levels suggesting that bDNA having miRNA sequences can selectively bind to mRNA and inhibit translation. Secondly, the downstream gene expression of p21 and p27 was also significantly downregulated in presence of miR-bDNA nanostructures. The cell proliferation activity was progressively increased in presence of miR-bDNA nanostructures which confirms the reduced tumor suppression activity of FOXO1 and the downstream gene expression. This finding can be explored to design novel bDNA structures which can downregulate the tumor suppressor proteins in normal cells and induce cell proliferation activity to identify early-phase markers of cancer.

**Supplementary Information:**

The online version contains supplementary material available at 10.1186/s12885-022-10423-8.

## Introduction

The FOXO transcription factors play a major role in regulating the gene expression of cell cycle progression, apoptosis, cell differentiation, stress response, DNA damage control, and vital cellular functions [1–3]. Further, FOXO1 is identified as a key tumor suppressor protein in breast cancer that regulates cell proliferation, invasion, metastasis and survival [4]. Activation of FOXO members promotes cell cycle arrests at G1/S stage by upregulating cell cycle inhibitors p27 and p21 [5, 6]. Thus, the downregulation of FOXO1 expression leads to dysregulation of cell cycle regulators which induce cell proliferation and play important role in the formation of cancer. Several study provides information regarding downregulated FOXO1 protein level in cancers, including glioblastoma [6], endometrial [7], ovarian carcinoma [8], prostate [9], and lung carcinoma [10]. Nevertheless, restoration of FOXO1 tumor suppressor protein in endometrial carcinoma leads to decrease in cell proliferation [7]. Further, Guttilla and White [11] demonstrated that the overexpression of FOXO1 strongly inhibits cell proliferation and induced apoptosis in breast cancer MCF7 cell line. Thus, the activation of FOXO1 is regarded as a therapeutic strategy for cancer.

Recently, microRNAs have been reported to modulate post-transcriptional regulation of mRNA resulting in the suppression of target gene expression [12, 13]. OncomiRNAs like miR-27a, miR-96, and miR-182 are upregulated in breast cancer and they collectively downregulate the expression of FOXO1 [12]. Thus, for the upregulation of tumor suppressor protein FOXO1, the oncomiRs need neutralization by antimiRNAs. Guttilla and White [11], demonstrated the downregulation of oncomiRs by administration of antimiRNAs and reported the overexpression of FOXO1 in MCF7 cell line. Recently, self-assembled branched DNA (bDNA) nanostructures have been evolved as economic and efficient strategy for miRNA-based cancer therapy [14–20]. Previously, our group has also reported the synergistic downregulation of oncomiRs and upregulation of FOXO1 by antimiR-bDNA nanostructures which selectively binds to the oncomiRs 27a, 96 and 182 in MCF-7 cell line [21].

In last two decades, substantial effort has been made for the delivery of antimiRs for upregulating tumor suppressor proteins or miRNA mimics for downregulating oncogenic proteins. Despite of restoring miRNAs and suppressing cancer cell proliferation using miRNA-based therapeutics very less attention has been made to initiate and activate cell proliferation in the presence of oncogenic miRNAs. Expression of oncogenic miRNA in normal cell is relatively low as compared to cancer cells. Once the oncogenic miRNAs and its target pathways are switched on in the normal cells they are transformed into cancer cells. Therefore, the transition from normal cell to cancer cell will be very useful in identifying early markers for cancer diagnostics. Keeping this as background, we hypothesize that if a group of oncogenic miRNAs can be delivered to cell lines they will collectively downregulate tumor suppressor protein and activate cell proliferation. To validate the idea we prefer to target transcription factor FOXO1, which is a major tumor suppressor protein and found to be downregulated in breast cancer by oncogenic miRNAs 27a, 96 and 182 [11, 21]. Despite substantial research on FOXO1 activity, the regulation of FOXO1 expression particularly in breast cancer is poorly understood. However, it has been hypothesized that downregulation of FOXO1 is a critical stage in the development of tumors. Nevertheless, researcher suggested that post-transcriptional mechanism, particularly regulation by miRNAs was responsible for the down-regulation of FOXO1 expression [11, 7]. Though, downregulation of FOXO1 is a pivotal step for forming tumorigenesis, the mechanism of initiation of tumorigenesis by down-regulated FOXO1 through exogenous miRNAs is yet to be understood. Keeping this as background, the present study demonstrates the role of exogenous oncomiRs on the expression of FOXO1 in breast cancer cell line and its effect on cancer cell proliferation. In the current study, self-assembled bDNA nanostructures has been explored to carry miR-27a, miR-96, miR-182 in the overhangs to downregulate the expression of FOXO1 in breast cancer MCF7 cell line. Thus, for the first time our data reports that bDNA nanostructure can carry oncogenic miRNA sequences and selectively recognize the target mRNA resulting into down-regulation of tumor suppressor protein and downstream cell cycle inhibitors p27 and p21in MCF7 cell line.

## Materials and methods

### Designing and self-assembly of bDNA nanostructures carrying microRNAs

**Fig. 1:**
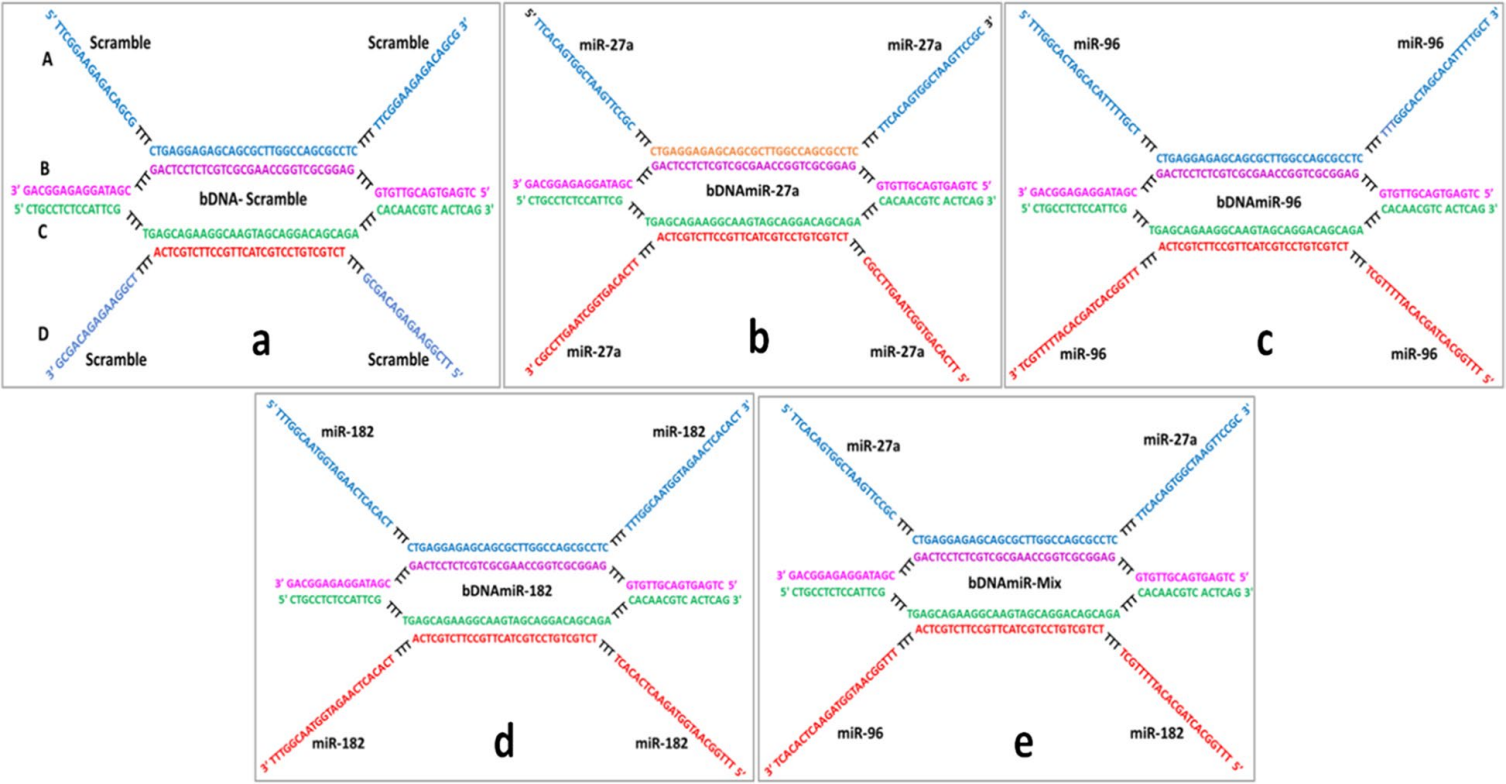
Schematic representation of self-assembled branched DNA (bDNA) nanostructures. bDNA nanostructures containing scramble sequence in the overhangs of bDNA-Scramble (**a**) miR-27a sequence in bDNAmiR-27a (**b**) miR-96 sequence in bDNAmiR-96 (**c**) miR-182 sequence in bDNAmiR-182 (**d**) and miR 27a, miR 96 and miR 182 in bDNAmiR-Mix nanostructures (**e**)

The designing of bDNA structures was performed as described previously [22, 23] and oligos were purchased from Integrated DNA Technology (IDT), USA without any modification or purification (Table S1). In brief, the bDNA monomeric structure composed of four oligonucleotides namely, strands A, B, C and D. The external region of strands B and C are complementary to each other, whereas the internal regions are complementary to strands A and D respectively (Fig. 1). Nevertheless, the overhangs of strands A and D are not complementary to each other or to any other oligonucleotides, thus they are replaced with either scramble sequences or miRNA sequences (Fig. 1). Since microRNAs 27a, 96, and 182 bind to the 3’ UTR of FOXO1 (Fig. S1), it prompted us to design the self-assembled miR-bDNA structures that can bind to the 3’ UTR of FOXO1. Thus, miR-bDNA nanostructures such as bDNAmiR-27a, bDNAmiR-96, bDNAmiR-182 are generated containing respective miRNA sequences in the overhangs (Table S2, Fig. 1). Similarly, bDNAmiR-Mix contains double dose of miR-27a and single dose of miR-96 and 182 for downregulating the mRNA and protein of FOXO1 whereas bDNA-Scramble is devoid of miRNA sequences (Fig. 1). 1 µM of each oligonucleotides were taken from a stock of 100 µM into 25 µl reaction mixture to prepare bDNA structures as mentioned earlier [21, 22].

### Characterization of bDNA nanostructures

The integrity of the self-assembled bDNA nanostructures was examined using 10% native polyacrylamide gel electrophoresis (nPAGE) as described previously [24]. The samples were electrophoresed for 2 h at 4°C in a vertical electrophoresis unit (SE260, Hofer, USA) at a constant voltage of 150 V by taking 1xTAE as running buffer. Then, the gels were stained with ethidium bromide solution (0.5 µg/ml) for 30 min and image was taken using FluroChem E system (Cell Biosciences).

The conformation of bDNA-miRs was examined using Circular Dichroism (CD) spectrophotometer (Chirascan, Applied Photophysics) as mentioned earlier [25]. The scan rate of spectra was recorded at 60 nm/sec with bandwidth of 1 nm and a time per point of 0.5 sec. All measurements were done at 25°C between 320 to 200 nm by using a quartz cuvette of 1 mm path length. Three spectra per sample were averaged for getting the final spectrum and was corrected against buffer as background.

### Serum stability and gel retardation assay

To study the stability of different bDNA-miR structures, 1 µM of bDNA was taken in 10% Fetal bovine serum (FBS). After mixing, the samples were incubated at 37°C for 0, 2, 4, 8, 12, 24 and 48 h. Similarly, free miRNA oligos were also examined for serum stability assay in presence of 10% FBS. Then the incubated products were analyzed by 1.5% agarose gel at 100 V for 30 min. The miRNA binding to 3’ UTR of FOXO1 was studied for binding scores using online miRNA target prediction tool TargetScan, Pictar, miRANDA (Fig. S1). Further, the integrity of microRNA binding to 3’UTR of FOXO1 region were evaluated using bDNA-miR and antimiR binding in gel retardation assay. AntimiR sequences were used to hybridize with corresponding bDNA-miRs *in vitro*. Different bDNA structures were incubated with respective antimiR sequence at 37°C for 2 h and then samples were run in 1.5% agarose gel at 100 V for 60 min.

### Cell Culture and transfection with bDNA nanostructures

The human breast cancer cell line MCF7 were obtained from the National Repository of Animal Cell Culture (NCCS Pune, Maharashtra, India). Cells were routinely maintained in Dulbecco’s Modified Eagles Medium (DMEM) (PAN Biotech, Germany) containing 10% (v/v) heat inactivated Fetal Bovine Serum (PAN Biotech, Germany) and 1% penicillin/streptomycin (PAN Biotech, Germany) in a humidified incubator with 95% humidity, 5% CO_2_ at 37°C (SANYO). Cells were seeded in 6-well tissue culture plates (corning) with density 3×10^5^ cells/well. When cells were grown up to 50–80% confluence, the cells were transfected with miRs and bDNAs. According to the manufacturers instruction (Invitrogen, USA) 3 µl of Lipofectamine 3000 was mixed properly with 122 µl Opti-MEM medium. For complex preparation, 100 nM of miR-27a, miR-96, miR-182, and miR-Mix or 25 nM of bDNA was mixed with 65 µl of Opti-MEM medium and 10 µl of P3000 reagent. Then, the diluted DNA was mixed with diluted Lipofectamine reagent in 1:1 ratio and incubated for 10 to 15 min at room temperature. After incubation, complexes were directly transfected to the cell line with final volume of 2 ml. The cells were divided into five groups such as bDNA-Scr, bDNAmiR-27a, bDNAmiR-96, bDNAmiR-182 and bDNAmiR-Mix for transfection study and incubated for 72 h. Similarly, groups of MCF7 cell line were also transfected with oncogenic miRs (miR-27a, miR-96, miR-182 and miR-Mix) as control to observe the declined expression of FOXO1.

### RNA isolation, cDNA synthesis and reverse transcription PCR

Total RNA was isolated from MCF7 cell line using miRNA Extraction Kit (217004, QIAGEN, USA) and reverse transcribed using cDNA synthesis kit (K1622, Thermo Scientific) according to manufacturer’s protocol and subsequently stored at -80°C. Reverse transcription of 2 µg RNA was performed using 1 µl of Oligo (dT)_18_ (0.5 µg/ µl), 4 µl of 5X Reaction buffer, 2 µl of 10 mM dNTPs, 1 µl of RiboLock RNase Inhibitor (20 U/µl), and 1 µl of RevertAid M-MuLV RT (200 U/µl) (Thermo Scientific). cDNAs were synthesized at 42°C for 1 h and terminated by heating at 70°C for 5 min. The reverse transcripts (cDNAs) were then subjected to PCR at 95°C for 3 min, denaturation at 94°C for 30 sec, annealing for 30 sec (57°C for 18S and p21, 57.4°C for FOXO1, 60°C for p27), extension at 72°C for 45 sec followed by the final extension at 72°C for 5 min. It is worthy to mention that 35 cycles for 18S, 36 cycles for FOXO1 and p27, 42 cycles for p21 were used for PCR. The 25 µl PCR reaction mixture contains 2.5 µl of 10X PCR buffer, 1.5 mM MgCl_2,_ 0.2 mM dNTPs, 25 pmol of each primer, 1 µl of cDNAs as template and 1U of Taq Polymerase (Thermo Scientific). The reverse transcription PCR (BioRad, USA) was performed using gene specific primer (Table S3) and 18S-rRNA was used as internal control to normalize the gene expression. The PCR products were electrophoresed on 2% agarose gel containing ethidium bromide. The relative expression change in gel images was quantified using ImageJ software.

### Western blotting analysis

Whole cell lysate was prepared after 72 h of transfection in 6 well culture plates using RIPA buffer containing protease inhibitor. Extracted proteins from transfected cells were electrophoresed on 10% SDS-PAGE and were subsequently transferred onto PVDF membrane in a constant voltage. The immunoblot was blocked in 5% skimmed milk powder for 1.5 h and incubated overnight with primary antibody FOXO1, p21, p27 and GAPDH under shaking condition at 4°C. All antibodies were procured from Cell Signaling Technology, (Danver, MA, US). Next day the blot was washed five times with Tris-buffered saline containing Tween-20 (TBST) and the membrane was then incubated with goat anti-rabbit secondary antibody conjugated with horseradish peroxidase (HRP) for 1 h at room temperature. After thorough ringing the membrane was developed using luminol on X-rays in the dark room.

### Cell viability assay

MTT (3-(4, 5-Dimethyl-2-thiazolyl)-2, 5-diphenyl-2H-tetrazolium bromide) assay was performed to study the cell viability in presence of bDNA structures. 3 x10^3^ numbers of cells were seeded on 96-well culture plates. The cells were transfected with five different bDNA structures after 24 h of seeding. After stipulated time point, 100 µl of MTT (5 mg/ml, Sigma, USA) was added to each well and incubated for 3 h at 37ºC. The growth medium containing MTT was removed and the formazan crystals were dissolved in 150 µl of dimethyl sulphoxide (DMSO, Sigma, USA). The plates were read at 570 nm (Microplate Reader, BioRad, USA) for absorbance and the viable cells were calculated.

### Statistical analysis

Each experiment was tested for three biological repeats. One way ANOVA was performed to test the statistical significance followed by Duncan’s multiple range test. A difference was considered statistically significant at *P* < 0.05.

## Results

### Characterization of self-assembled bDNA nanostructures

**Fig. 2:**
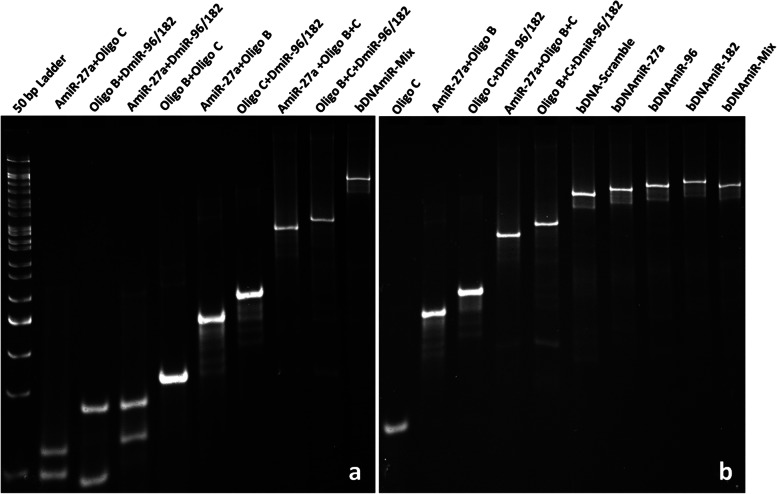
Characterization of self-assembled bDNA nanostructures. Gel image display the binding of oligonucleotides to form di-oligo and tri-oligo complexes and bDNAmiR-Mix structures containing the sequence of miR-27a, miR-96, miR-182 in the four overhangs. Sample composition in each lane is mentioned in top of the lane. bDNAmiR-Mix shows decrease electrophoretic mobility with respect to di and tri-oligo complexes formation in 10% nPAGE (**a**). Migration of bDNA structures (bDNAmiR-27a, bDNAmiR-96, bDNAmiR-182, bDNAmiR-Mix) having miR sequences in overhangs. bDNA structures (lane 6 to 10) shows less electrophoretic mobility with respect to mono, di and tri-oligo complexes. The single bands in each lane indicates sequence-specific base pairing among oligonucleotides (**b**)

The intensity of individual oligonucleotide showed a clear band whereas the complementary oligonucleotides (AB, BC, CD) were forming di-oligo complexes exhibiting decreased electrophoretic mobility (Fig. 2a). The di-oligos, tri-oligos and bDNA-scramble have different electrophoretic mobility suggesting migration of nucleic acids in PAGE is dependent on size, shape, and conformation (Fig. S2a,b,c). Similarly, the bDNAmiR-27a, bDNAmiR-96, bDNAmiR-182, and bDNAmiR-Mix were characterized with a clear intense band suggesting the formation of stable unimolecular bDNA structures (Fig. 2b). Nevertheless, all the self-assembled bDNA structures showed B-form of DNA with characteristic positive peaks at ~ 280 nm and ~ 220 nm whereas a negative peak at ~ 250 nm (Fig. S3).

### Stability of miR-bDNA and selective binding to mRNA

Nuclease stability of bDNA nanostructures was examined in presence of *FBS* which comprises ~ 256 U/L of DNase 1. After incubation at 37°C the relative intensity of miR-bDNA was found to be stable for 12 h then slowly decreases upto 48 h (Fig. S4). On the contrary, naked miRNAs are unstable in presence of nuclease. The gel retardation assay reveals a significant retardation of DNA suggesting the sequence specific binding between miR-bDNA and FOXO1 mRNA (Fig. S5).

### Expression of FOXO1, p21, and p27 in response to miRNA

**Fig. 3:**
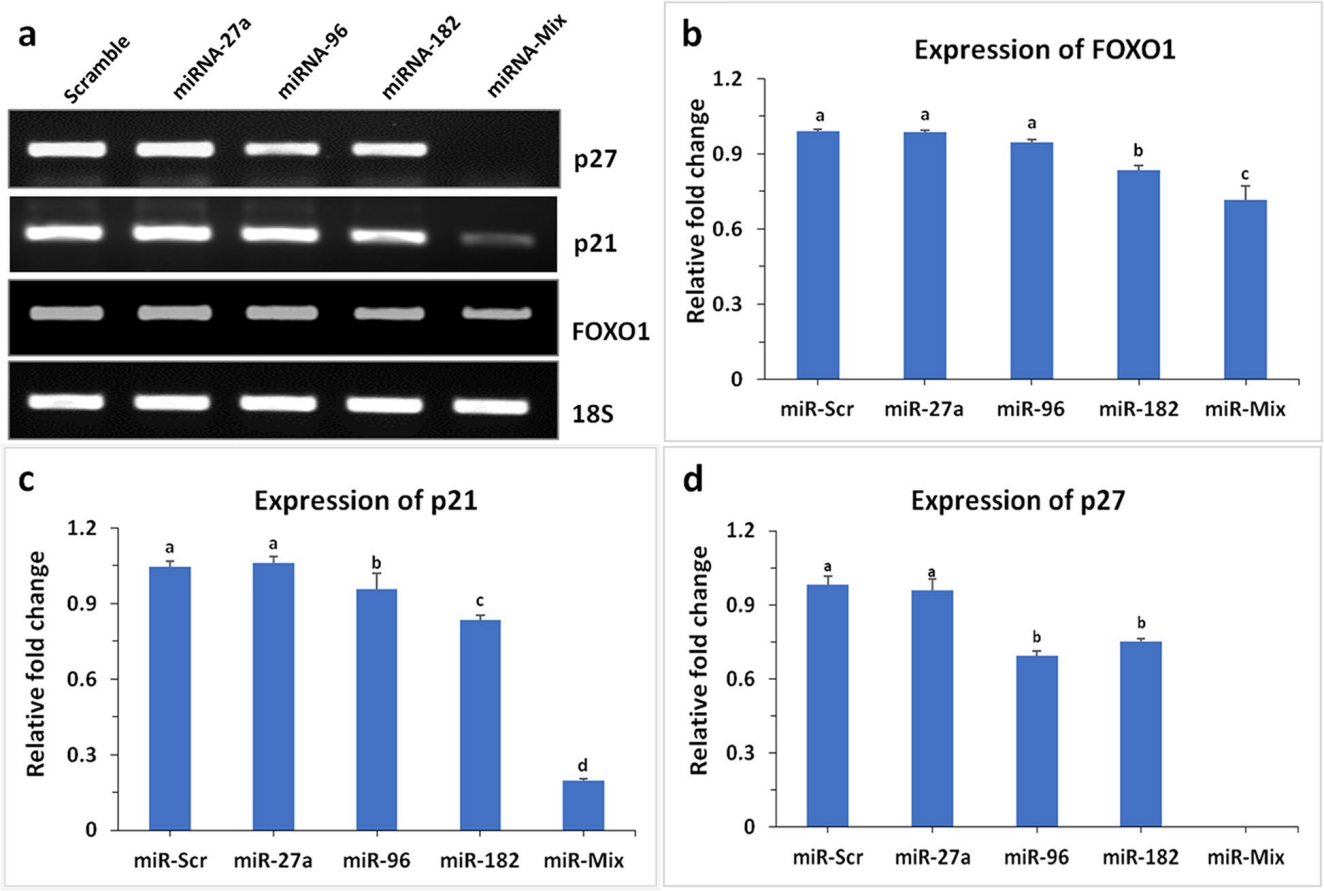
Gene expression in response to transfection of miRNAs. RT-PCR products resolved in agarose gel showing the expression of 18S, FOXO1, p21, and p27 after transfected with miRs (**a**). Relative expression of FOXO1 (**b**) p21 (**c**) and p27 (**d**) on breast cancer cell lines transfected with miRs was quantified using ImageJ software against 18S as quantitative control. One way ANOVA was used for statistical significance at *P *< 0.05

A significant (*P* < 0.001) decreased expression of FOXO1 was observed in miR-182 transfected cells whereas, no change in expression was noticed with miR-27a and 96 (Fig. 3a,b). Interestingly the downregulation of FOXO1 was further decreased by miR-Mix (Fig. 3a,b). Further, the expression of well-known CDK inhibitors like p21 and p27 was examined in response to miRNAs. A significant reduction in expression of p21 was evident in cells transfected with miR-96 (*P* < 0.05) and miR-182 (*P* < 0.001) (Fig. 3c). The expression was further reduced (*P* < 0.001) when cells were transfected with miR-Mix (Fig. 3c). On the other hand, the expression of p27 was significantly (*P* < 0.001) repressed in presence of miR-96 and miR-182 and disappeared with miR-Mix (Fig. 3d).

### miRNA-bDNA modulates the expression profile of FOXO1, p21, and p27

**Fig. 4:**
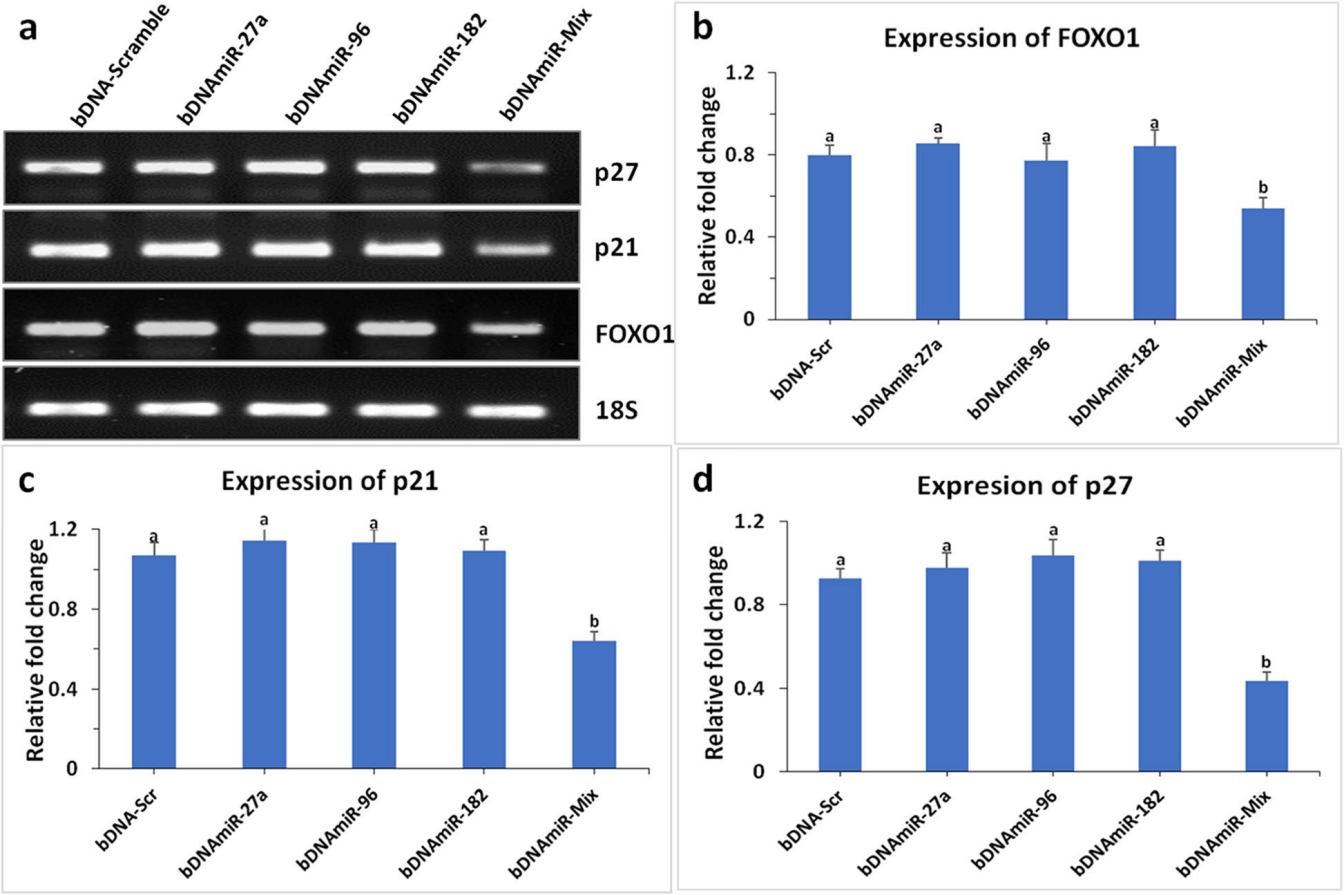
Transcript profile after transfection with bDNAmiR nanostructures. Agarose gel shows the transcripts level of FOXO1, p21, p27 and 18S in response to the transfection with bDNA-miRs (**a**). Relative expression change of FOXO1 (**b**) p21 (**c**) and p27 (**d**) on breast cancer cell line quantified using ImageJ software with 18S as quantitative control. One way ANOVA was used for statistical significance at *P* < 0.01

On the contrary, no significant change in expression of *FOXO1, p21, and p27* was found in cells transfected with bDNAmiR-27a, bDNAmiR-96 or bDNAmiR-182 (Fig. 4). However, a significant (*P* < 0.01) decrease in FOXO1 expression was evident when transfected with bDNAmiR-Mix (Fig. 4b). Nearly 40% downregulation of FOXO1 was observed as compared to bDNA-Scr (Fig. 4a, b). Similarly, no significant change was observed in the expression of p21 and p27 in the presence of miR-bDNA nanostructures (Fig. 4c,d). Nevertheless, a significant (*P* < 0.001) decrease in expression of p21 and p27 was noticed while transfected with bDNAmiR-Mix (Fig. 4a,d).

### Downregulation of FOXO1 protein and downstream gene expression

The target site of miR-27a, miR-96, miR-182 on the 3’ UTR of FOXO1 has been confirmed using TargetScan, Pictar, and miRanda and found to contain six positions for binding to miRNAs (Fig. S1). Thus, to examine the influence of bDNA-miRs on FOXO1, the endogenous expression of FOXO1 protein was evaluated using western blot analysis (Fig. S6). In comparison to bDNA-Scr, significant decrease in FOXO1 protein level was noticed when transfected with bDNAmiR-27a, and bDNAmiR-Mix. However, an unaltered level of FOXO1 was observed in bDNAmiR-96 and an enhanced level was evident in cells transfected with bDNAmiR-182. FOXO1 is known to regulate the target gene expression of p21 and p27. Interestingly, the protein level of p21 and p27 was observed to be increased in all the bDNA-miRs transfected samples as compared to bDNA-Scr (Fig. S6).

### Effect on cell proliferation by miR-bDNA nanostructures

**Fig. 5:**
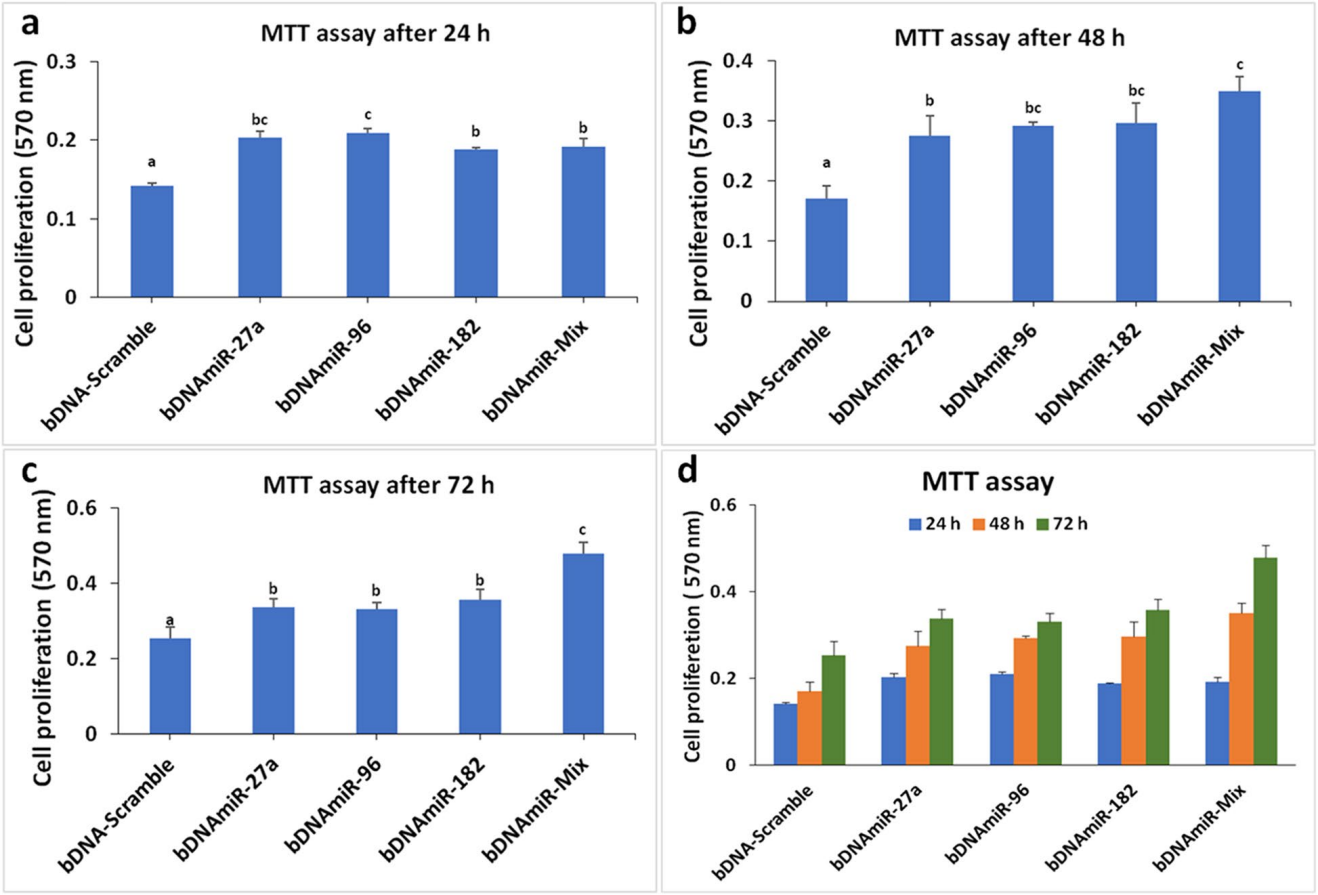
Enhanced cell proliferation of MCF7 breast cancer cell line in presence of bDNAmiR-Mix. MTT assay revealed the effect of bDNAmiRs on breast cancer cell growth after 24h, 48h and 72h in MCF cell line. One way ANOVA is used for statistical significance at *P* < 0.001

Cell proliferation was monitored in different time interval from 24 h, 48 h and 72 h. A significant (*p*<0.001) increase in cell proliferation was found with bDNAmiR-27a, bDNAmiR-96, bDNAmiR-182 and bDNAmiR-Mix as compared to bDNA-Scr after 24 h of incubation. Similarly, higher cell proliferation was noticed after 48 and 72 h of incubation with all bDNA nanostructures. Importantly, highest cell proliferation was noticed in MCF7 cells transfected with bDNAmiR-Mix structures (Fig. 5).

## Discussion

Breast cancer is the second leading cause of cancer death among women after lung cancer in the United States. It is estimated that 2,68,600 cases diagnosed with invasive breast cancer in the United States and annually 41,760 patients die in breast cancer [26]. Thus, the molecular mechanism of breast cancer development and progression deserve detailed investigations. FOXO1 is a master regulator that regulates cell cycle proliferation, invasion, metastasis and in several cancers including breast cancer [3]. Now it is well evident that miRNAs play a vital role in proliferation and metastasis of cancer in general and post-transcriptional regulation of tumor suppressor mRNAs in particular [12]. Currently, miR-27a, miR-96, and miR-182 are known oncomiRs in breast cancer which coordinately reduce the expression of FOXO1 [11]. Nevertheless, in variety of other cancer miR-27a, miR-96, and miR-182 act as oncomiRs to suppress FOXO1, including renal cell cancer [27], bladder cancer [28], Prostate cancer [29], colorectal cancer [30], thyroid carcinoma [31], ovarian cancer [32], liver cancer [33], gastric cancer [34], adenocarcinoma [35], and cervical cancer [36]. Thus, understanding the regulation of FOXO1 expression is vital in managing and monitoring the cancer in general and breast cancer in particular. Moreover, these findings strongly support that miR-27a, miR-96 and miR-182 act as oncogenic miRNAs for downregulating FOXO1 and cell cycle proliferation. We have reported previously that error-free hybridization between primers and its complementary sequences act as the driving force for self-assembly of bDNA structure [22]. Herein, we have replaced the overhangs of bDNA structures with respective miRNAs to form bDNAmiR-27s, 96, 182 and in case of bDNAmiR-Mix, four overhangs contain two doses of miR-27a and one dose of miR-96 and miR-182 each. Whereas, bDNA-Scramble is devoid of miRNA sequences in the overhangs. The advantage of bDNA nanostructure is to carry multiple miRNAs to deliver in one go and in one location as compared to single stranded miRNAs. Nevertheless, application of antimiRNAs 27a, 96, and 182 down- regulate the expression of oncogenic miRNAs in MCF7 and synergistically upregulate the expression of FOXO1 [11, 21]. However, the cellular response and FOXO1 expression was yet to be investigated when cells are subjected to the simultaneous transfection of multiple oncogenic miRNAs. Secondly, these model oncogenic miRNAs to be transfected to normal cell lines to initiate cell proliferation and find early diagnostic marker for cancer. Since self-assembled bDNA nanostructures are biocompatible drug carrier, bDNAs were designed to carry miR-27a, miR-96, miR-182 and transected to MCF7 cell line for understanding the cell proliferation and FOXO1 expression. As expected, a significant decrease in FOXO1 expression was noticed in MCF7 while transfected with bDNA nanostructures. The molecular interaction between bDNA nanostructure and FOXO1 mRNA also caused translational inhibition of FOXO1. Further, FOXO1 expression was significantly decreased by miR-182 and miR-Mix transfection. Nevertheless, miR-Mix or bDNAmiR-Mix works better than individual miRNAs which supports earlier data on the multiple miRNA regulation to a particular gene expression [21]. However, no significant variation was noticed when cells transfected with bDNAmiR-27a, bDNAmiR-96 and bDNAmiR-182. Possibly, indigenous expressions of miRNAs have occupied much of the binding position of the targeted mRNA. Nevertheless, a significant downregulation of mRNA was observed by the bDNAmiR-Mix as compared with the respective bDNAmiRs.

The expression of p21 and p27 was significantly repressed when cells transfected with miR-96, miR-182 and miR-Mix. But in case of miR-27a and 96 transfections to the cells there is no such change was observed in the expression of FOXO1. The results suggest that expression of p21 and p27 is largely dependent on miRNAs-mediated downregulation of FOXO1. The endogenous FOXO1 protein decreases when cells were transfected with bDNAmiR-Mix. Thus, the downregulated translated product of FOXO1 by bDNAmiR nanostructures support the hypothesis that FOXO1 protein expression is downregulated at the post-transcriptional level by multiple miRNAs. The expression of p21 and p27 protein was observed to be increased in all the bDNA-miRs transfected samples. Possibly, the expression of p21 and p27 was regulated by other miRNAs.Nevertheless, miR-27a and miR-96 mediate cell proliferation by regulating cyclin D1, p21 and p27 [37, 38]. In addition, miR-27a, miR-96 and miR-182 could promote migration and invasion by the regulation of PTPN9 in breast cancer [39], and PDCD4 in hepatocellular cancer [40]. Since overexpression of FOXO1 is associated with decreased proliferation and colony size in MCF7 cells, we expect increased cellular proliferation in MCF7 cells transfected with the miR-bDNA nanostructures. Previously some studies demonstrated that miR-27a, 96 and 182 co-ordinately downregulate the FOXO1 protein level, indicating these microRNAs co-ordinately regulate endogenous FOXO1 protein level [11]. These findings corroborate our hypothesis that transfection of a combination of miRNAs results in enhanced cell proliferation and post-transcriptional repression of FOXO1 expression. Therefore, cellular proliferation was analyzed in MCF7 cells transfected with bDNA-miRs at different incubation periods. A significant cell proliferation was noticed even after 24h of incubation. However, higher cell proliferation was noticed with bDNAmiR-Mix nanostructures which suggest the synergistic effect of oncogenic miRNAs on cell proliferation. Secondly, these findings clearly suggest that miR-27a, miR-96 and mR-182 promote cell proliferation by targeting not only FOXO1 but also other tumor suppressor genes. However, to validate our hypothesis further experiments to be carried out on *in vivo* models. Nevertheless, bDNA nanostructures have remarkable advantages for biomedical application due to biocompatible, biodegradable and non-toxic biomaterials.

## Conclusions

In conclusion, we have designed and synthesized self-assembled bDNA nanostructures bearing microRNA sequences as functional unit in all four overhangs that target intracellular tumor suppressor gene FOXO1 in MCF7 breast cancer cell line. For the first-time the current communication reports the delivery of multiple miRNAs through self-assembled bDNA nanostructures and significant downregulation of FOXO1. Nevertheless, the enhanced cell proliferation by bDNA nanostructures is not only due to downregulation of FOXO1 but also associated tumor suppressor proteins. Thus, the model oncogenic miRNAs like miR-27a, miR-96 and miR-182 can be transfected using bDNA nanostructures to various cell lines for switching cell proliferation and identifying early diagnostic markers for cancer. Nevertheless, the proof of principle can be extended to multiple oncogenic miRNAs which can be transfected to the cell lines and coordinately downregulate tumor suppressor proteins to study cell proliferation, invasion, and metastasis. Our findings in this study suggest that bDNA nanostructures can serve as nucleic acid therapeutics and advanced drug delivery carriers in the recent future.

## Supplementary Information

**Additional file 1:** **Table S1. **Oligonucleotide sequences used for preparation of self-assembled branched DNA nanostructures. **Table S2. **Designing of different self-assembled bDNA structures (bDNAmiR-27a, bDNAmiR-96, bDNAmiR-182 and bDNAmiR-Mix) having target sequence to 3’ UTR of FOXO1 mRNA. **TableS3. ** Primer sequences used for gene expression study. **Fig. S1.** Image showing the predicted binding sites of 3’ UTR of FOXO1 by miR-27a, miR-96 and miR-182. **Fig. S2. **Characterization of self-assembled bDNA structures. Gel image shows the intensity and integrity of single and di-oligo complexes in 10% nPAGE. Non complementary oligos shows no interaction with each other which revealed specificities of oligo designing, whereas oligos with complementary sequence results to form a desired di-oligo product (a). Characterization of single, di and tri-oligo complexes to form self-assembled bDNA-scramble in 10% nPAGE (b). Gel image showing the formation of bDNA-Scramble through the di-oligo and tri-oligo in 10% nPAGE (c). Formation of bDNA-Mix structures containing miR-27a, miR-96, miR-182 sequences in the four overhangs (d). Sample composition of each lane is mentioned on top of each lane. bDNAmiR-Mix shows decrease electrophoretic mobility with respect to di and tri-oligo complexes.The single bands in each lane indicates precise base pairing among oligos. **Fig. S3.** Conformation of bDNA using Circular dichroism study. A typical right-handed stable conformation was noticed inall the self-assembled bDNA with a characteristic of signature positive peaks at ~280 nm and ~ 220 nm and negative peak at ~250 nm. **Fig. S4. **Serum stability assay of bDNA nanostructures. Agarose gel image display the stability of bDNA structure after incubation at 37°C for 0 to 48 h. (SFM: Serum free media, SSM: Serum supplemented media). **Fig. S5. **Gel retardation assay showing *in vitro* binding between bDNA-miR and its complementary antimiR sequences. Gel image showing the migration of bDNA in the absence (lane 1-5), and presence of antimiRs (7-10). A clear shift of band was seen in lanes 7 to 10 due to the binding between bDNA-miR and antimiR. In bDNA-Scr (lane 6) no change in migration was observed. **Fig. S6.** Western blot analysis of FOXO1 Expression. Endogenous expression of FOXO1, p21, p27 levels presented after transfection with bDNA-Scr and bDNA-miR (bDNAmiR-27a, bDNAmiR-96, bDNAmiR-182 and bDNAmiR-Mix) to breast cancer MCF7cell line. GAPDH was used as loading control.

## Data Availability

The datasets generated and/or analysed during the current study does not contain any data that needs to be submitted in any database, thus given in materials and methods section of the manuscript. However, all the data are available from the corresponding author on reasonable request.
