# Supplementary material for: Self-assembled DNA nanostructure containing oncogenic miRNA-mediated cell proliferation by downregulation of FOXO1 expression
Source: BMC Cancer. 2022 Dec 20;22:1332. doi: 10.1186/s12885-022-10423-8 (PMC9764560; doi:10.1186/s12885-022-10423-8)
Supplement: Supplementary file 1 — Additional file 1: Table S1. Oligonucleotide sequences used for preparation of self-assembled branched DNA nanostructures. Table S2. Designing of different self-assembled bDNA structures (bDNAmiR-27a, bDNAmiR-96, bDNAmiR-182 and bDNAmiR-Mix) having target sequence to 3’ UTR of FOXO1 mRNA. TableS3. Primer sequences used for gene expression study. Fig. S1. Image showing the predicted binding sites of 3’ UTR of FOXO1 by miR-27a, miR-96 and miR-182. Fig. S2. Characterization of self-assembled bDNA structures. Gel image shows the intensity and integrity of single and di-oligo complexes in 10% nPAGE. Non complementary oligos shows no interaction with each other which revealed specificities of oligo designing, whereas oligos with complementary sequence results to form a desired di-oligo product (a). Characterization of single, di and tri-oligo complexes to form self-assembled bDNA-scramble in 10% nPAGE (b). Gel image showing the formation of bDNA-Scramble through the di-oligo and tri-oligo in 10% nPAGE (c). Formation of bDNA-Mix structures containing miR-27a, miR-96, miR-182 sequences in the four overhangs (d). Sample composition of each lane is mentioned on top of each lane. bDNAmiR-Mix shows decrease electrophoretic mobility with respect to di and tri-oligo complexes.The single bands in each lane indicates precise base pairing among oligos. Fig. S3. Conformation of bDNA using Circular dichroism study. A typical right-handed stable conformation was noticed inall the self-assembled bDNA with a characteristic of signature positive peaks at ~280 nm and ~ 220 nm and negative peak at ~250 nm. Fig. S4. Serum stability assay of bDNA nanostructures. Agarose gel image display the stability of bDNA structure after incubation at 37°C for 0 to 48 h. (SFM: Serum free media, SSM: Serum supplemented media). Fig. S5. Gel retardation assay showing in vitro binding between bDNA-miR and its complementary antimiR sequences. Gel image showing the migration of bDNA in the absence (lane 1-5), a [file 12885_2022_10423_MOESM1_ESM.docx]

**Supplementary Information**

**Self-assembled DNA nanostructure containing oncogenic miRNA-mediated cell proliferation by downregulation of FOXO1 expression**

**Avishek Kar^1,2^, Kanchan Kumari^1,3^, Sandip K. Mishra^4^, Umakanta Subudhi^1,2,^***

^1^DNA Nanotechnology & Application Laboratory, CSIR-Institute of Minerals & Materials Technology, Bhubaneswar, 751013, India.

^2^Academy of Scientific & Innovative Research (AcSIR), Ghaziabad-201002, Uttar Pradesh, India.

^3^Department of Molecular Biology, Umea University, Sweden.

^4^Cancer Biology Laboratory, Institute of Life Sciences, Bhubaneswar, 751023, India.

* Corresponding author

**Table S1.** Oligonucleotide sequences used for preparation of self-assembled branched DNA nanostructures.


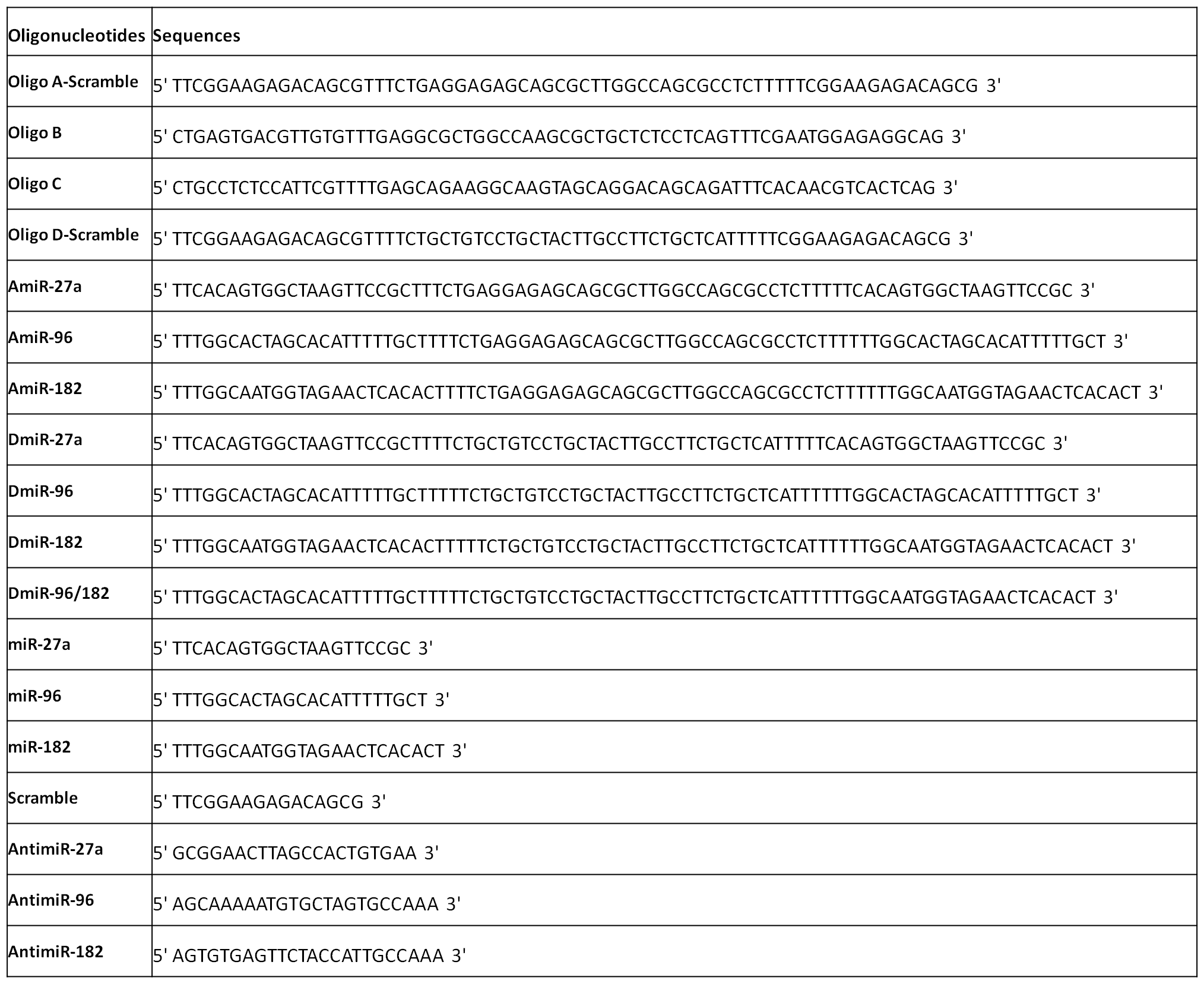


**Table S2.**  Designing of different self-assembled bDNA structures (bDNAmiR-27a, bDNAmiR-96, bDNAmiR-182 and bDNA-Mix) having target sequence to 3’ UTR of FOXO1 mRNA.


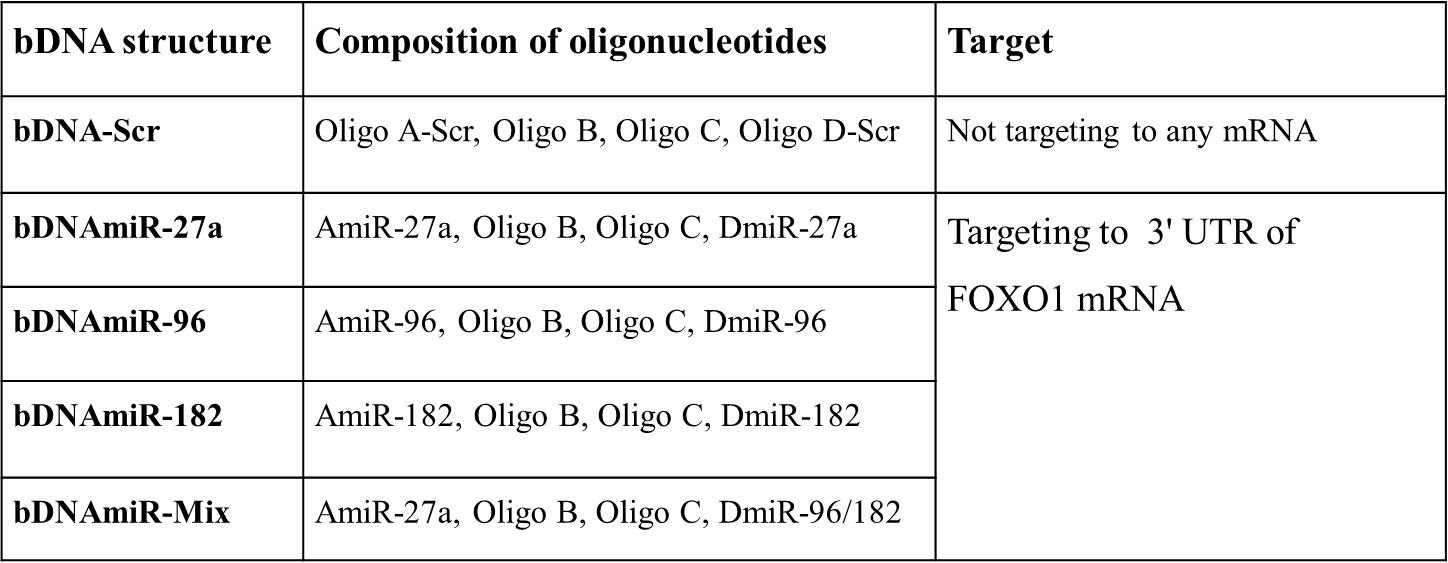


**Table S3.**  Primer sequences used for gene expression study.


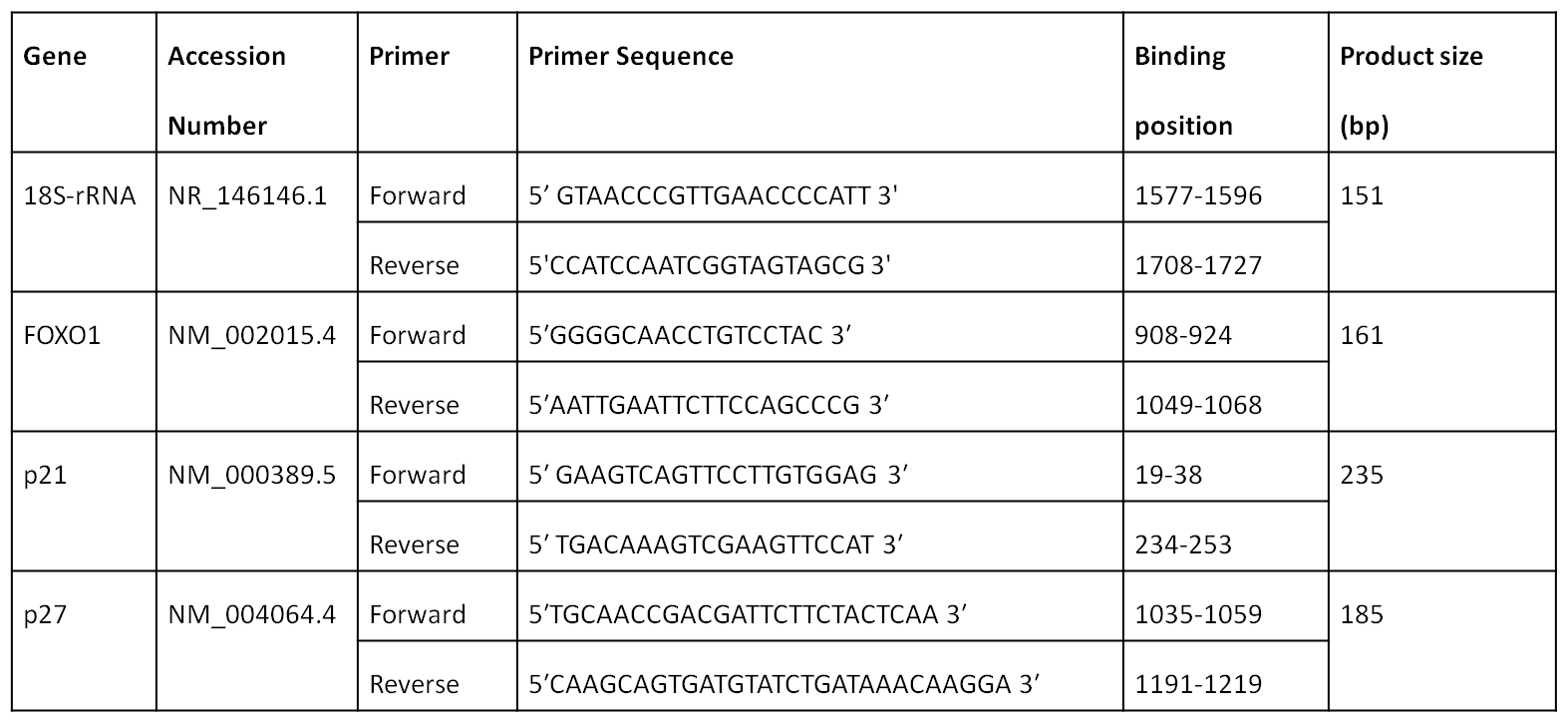


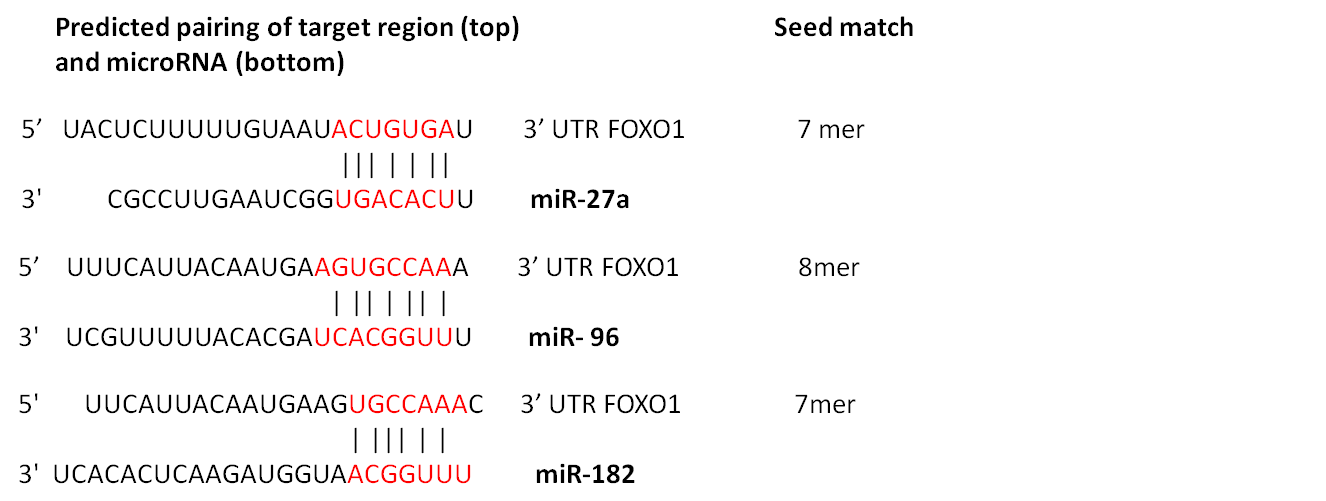


**Fig. S1** Image showing the predicted binding sites of 3’ UTR of FOXO1 by miR-27a, miR-96 and miR-182.


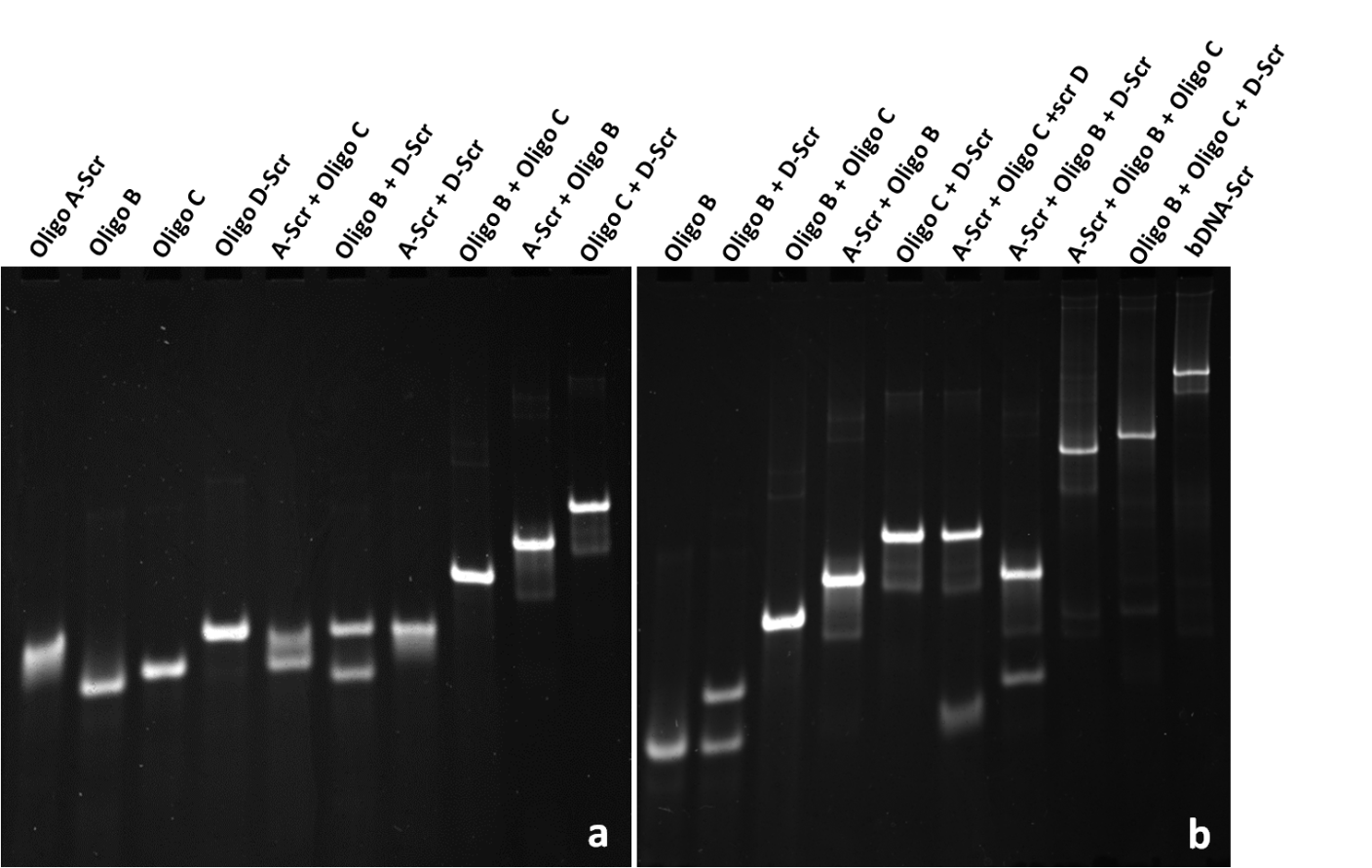


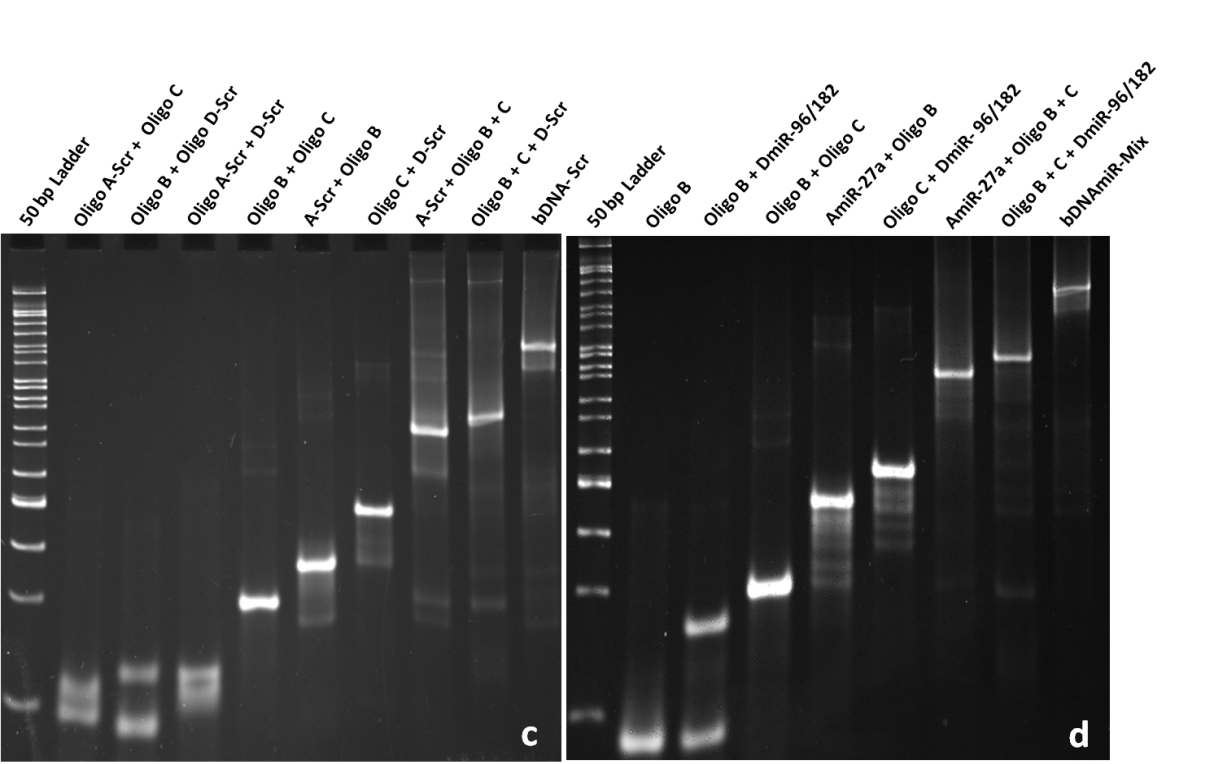


**Fig. S2 Characterization of self-assembled bDNA structures.** Gel image shows the intensity and integrity of single and di-oligo complexes in 10% nPAGE. Non complementary oligos shows no interaction with each other which revealed specificities of oligo designing, whereas oligos with complementary sequence results to form a desired di-oligo product (a). Characterization of single, di and tri-oligo complexes to form self-assembled bDNA-scramble in 10% nPAGE (b). Gel image showing the formation of bDNA-Scramble through the di-oligo and tri-oligo in 10% nPAGE (c). Formation of bDNA-Mix structures containing miR-27a, miR-96, miR-182 sequences in the four overhangs (d). Sample composition of each lane is mentioned on top of each lane. bDNA-Mix shows decrease electrophoretic mobility with respect to di and tri-oligo complexes. The single bands in each lane indicates precise base pairing among oligos.


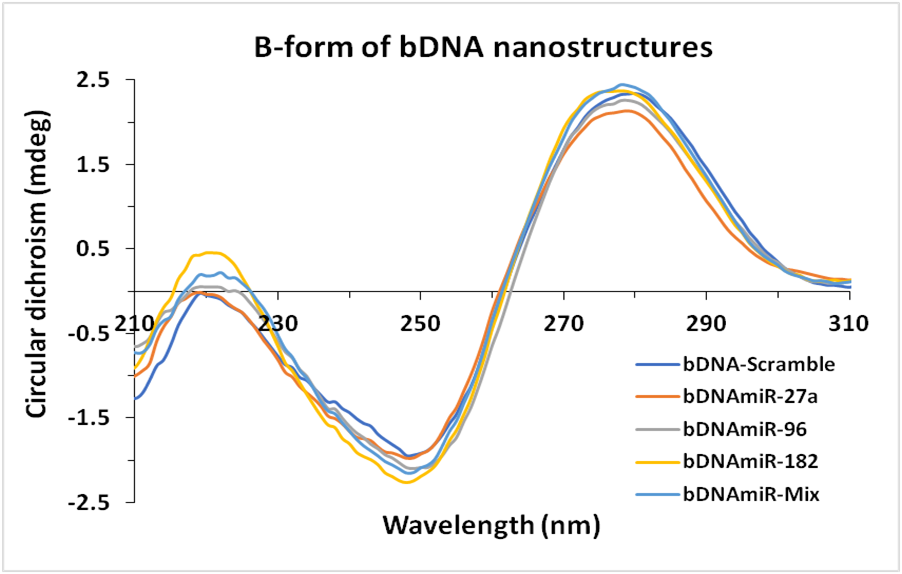


**Fig. S3** **Conformation of bDNA using** **Circular dichroism study**. A typical right-handed stable conformation was noticed in all the self-assembled bDNA with a characteristic of signature positive peaks at ~280 nm and ~ 220 nm and negative peak at ~250 nm.


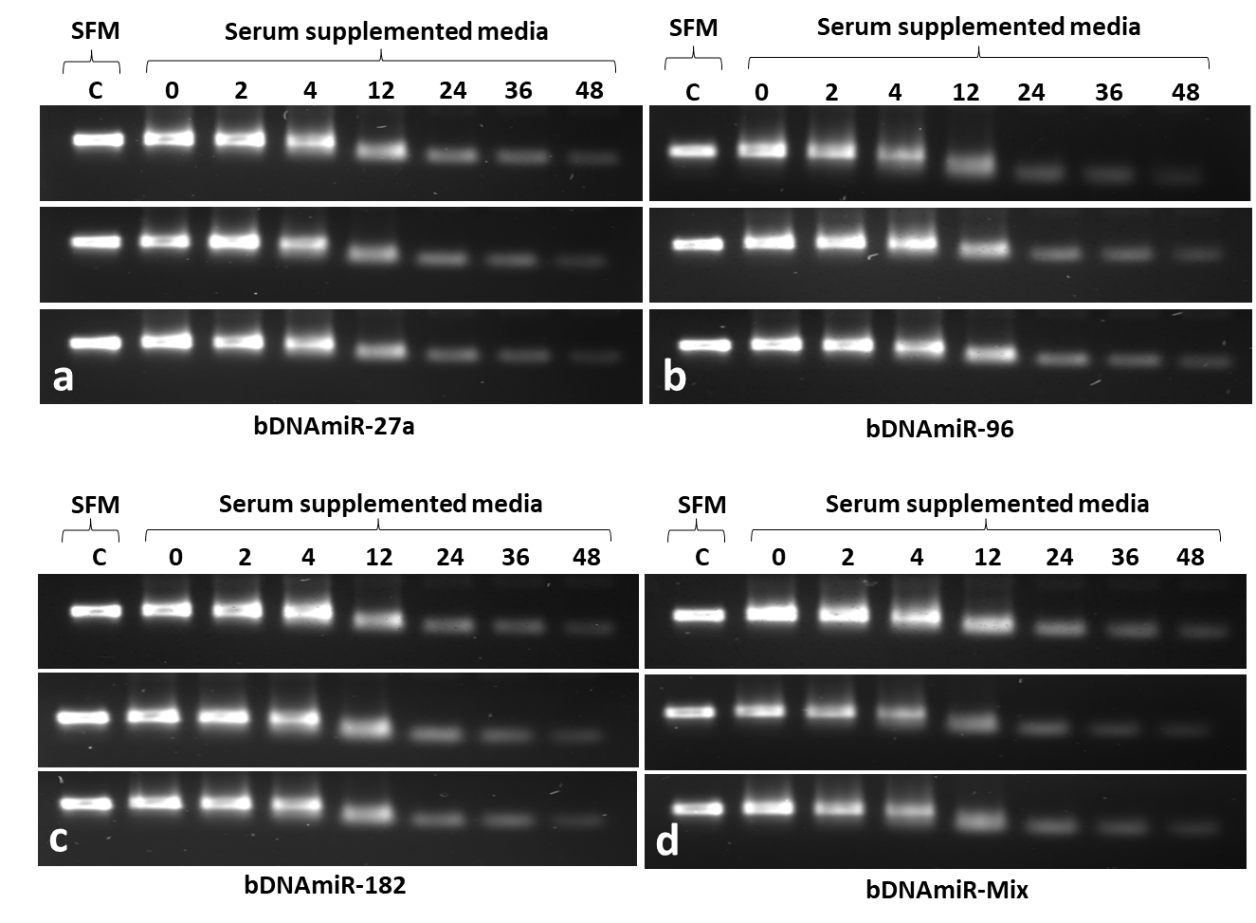


**Fig. S4 Serum stability assay of bDNA nanostructures.** Agarose gel image display the stability of bDNA structure after incubation at 37°C for 0 to 48 h. (SFM: Serum free media, SSM: Serum supplemented media).


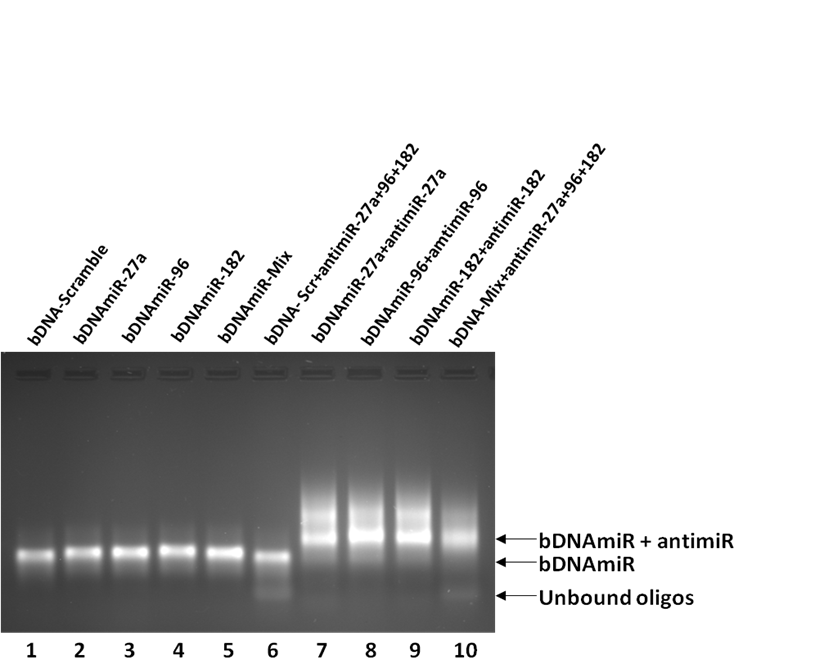


**Fig. S5 Gel retardation assay showing *in vitro* binding between bDNA-miR and its complementary antimiR sequences.** Gel image showing the migration of bDNA in the absence (lane 1-5), and presence of antimiRs (7-10). A clear shift of band was seen in lanes 7 to 10 due to the binding between bDNA-miR and antimiR. In bDNA-Scr (lane 6) no change in migration was observed.


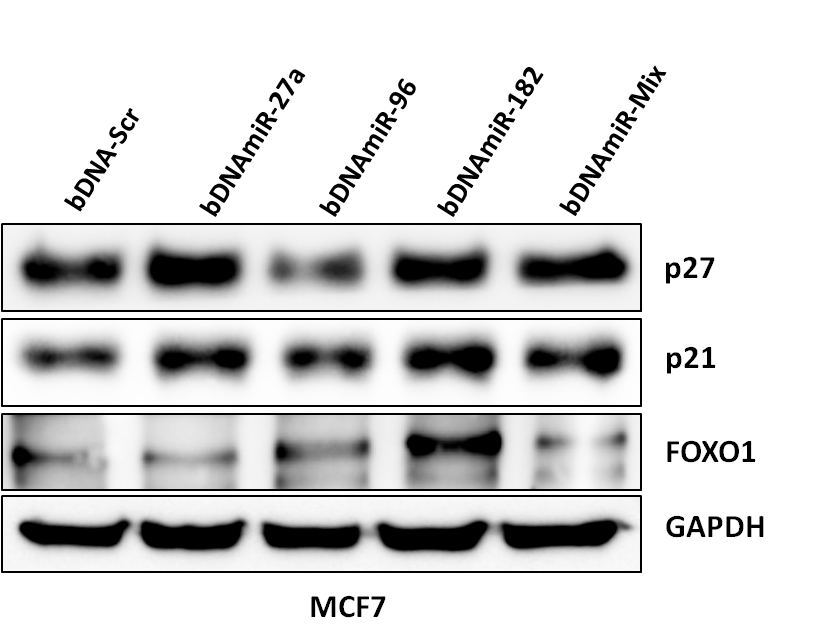


**Fig. S6** **Western blot analysis of FOXO1 Expression.** Endogenous expression of FOXO1, p21, p27 levels presented after transfection with bDNA-Scr and bDNA-miR (bDNAmiR-27a, bDNAmiR-96, bDNAmiR-182 and bDNA-Mix) to breast cancer MCF7 cell lines. GAPDH was used as loading control.

**Full length Gel Images**

**Part A: Full length gel images for main manuscript Figures**

**
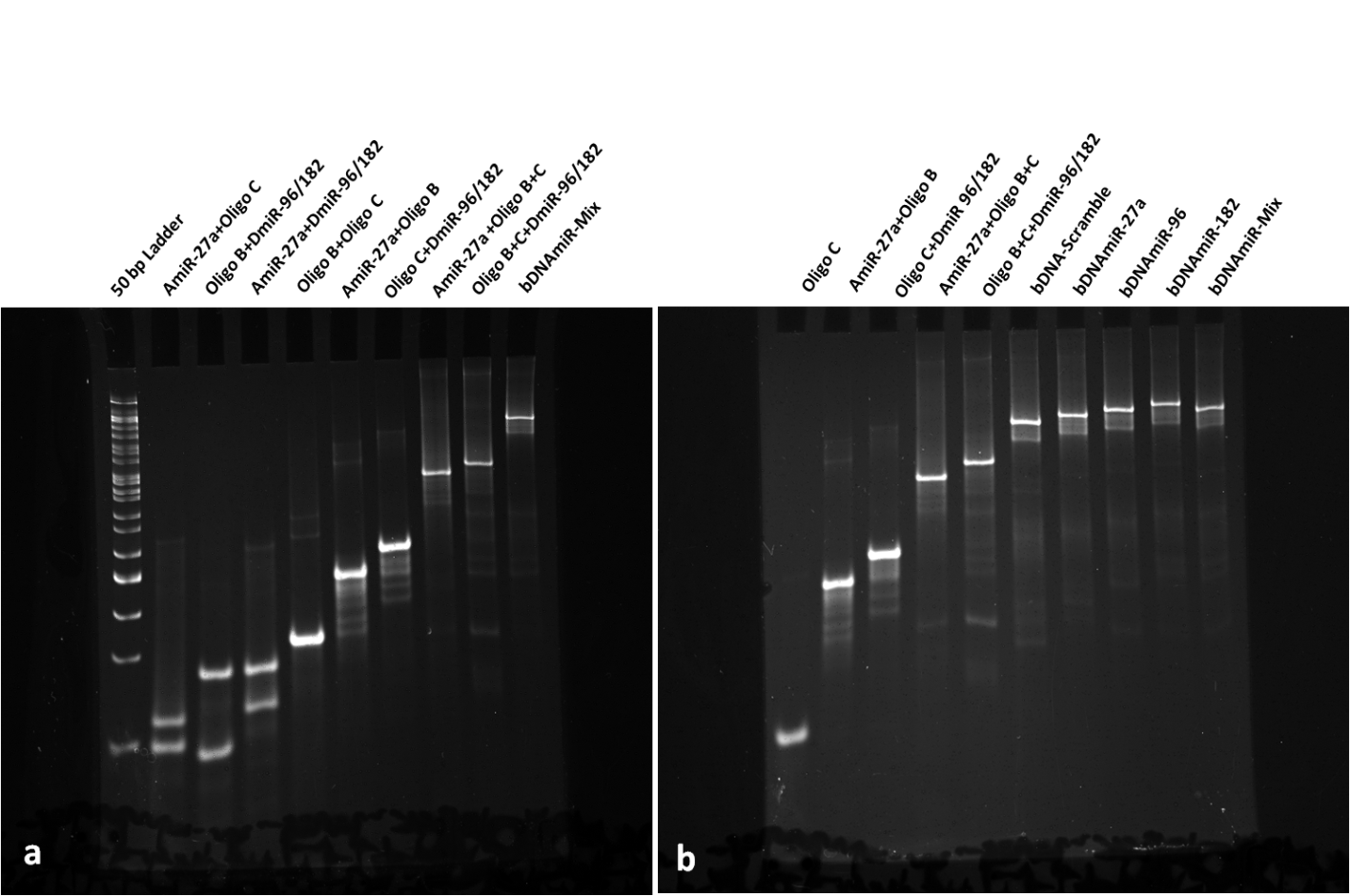
**

**Fig. 2 Characterization of self-assembled bDNA nanostructures.** Gel image display the binding of oligonucleotides to form dioligo and trioligo complexes and bDNAmiR-Mix structures containing the sequence of miR-27a, miR-96, miR-182 in the four overhangs. Sample composition in each lane is mentioned in top of the lane. bDNAmiR-Mix shows decrease electrophoretic mobility with respect to di and tri-oligo complexes formation in 10% nPAGE (a). Migration of bDNA structures (bDNAmiR-27a, bDNAmiR-96, bDNAmiR-182, bDNAmiR-Mix) having miR sequences in overhangs. bDNA structures (lane 6 to 10) shows less electrophoretic mobility with respect to mono, di and tri-oligo complexes. The single bands in each lane indicates sequence-specific base pairing among oligonucleotides (b).

**
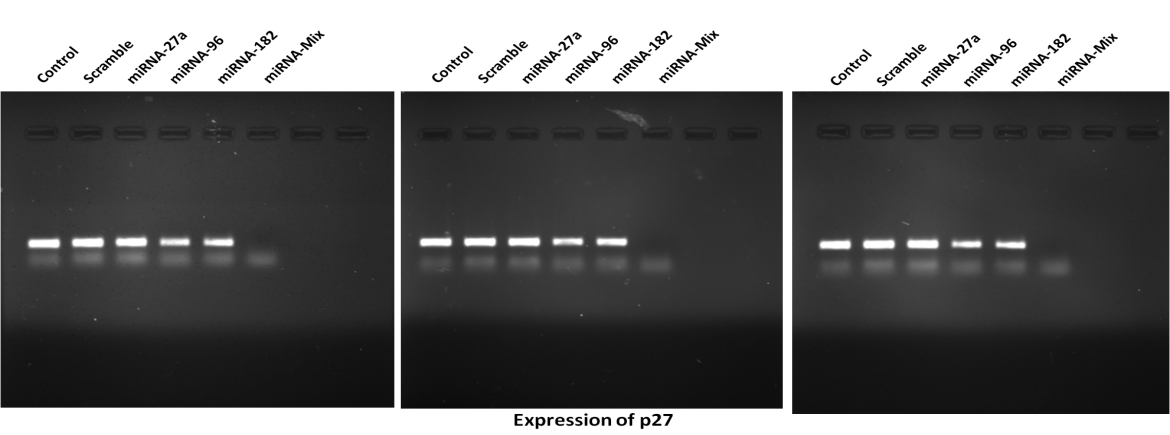

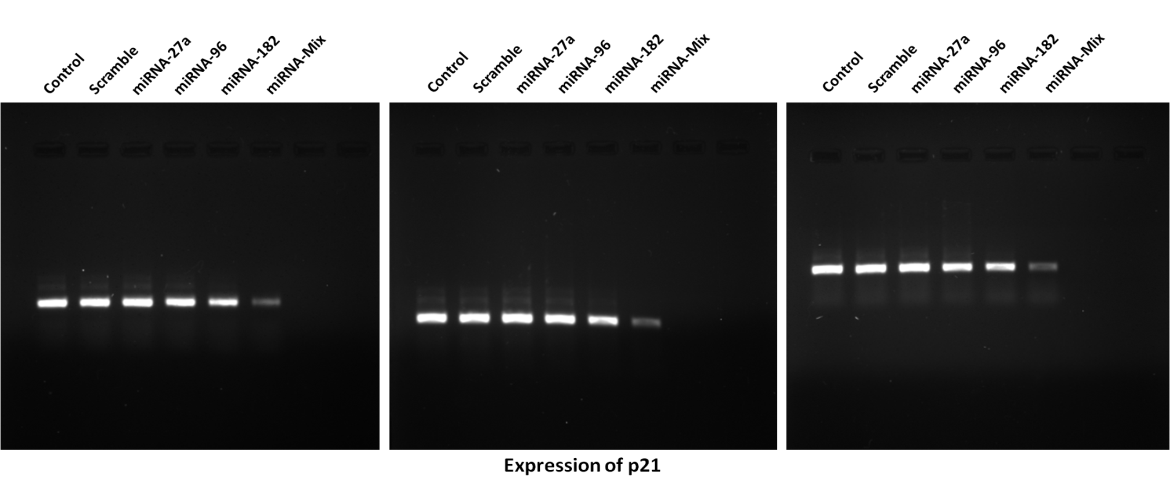

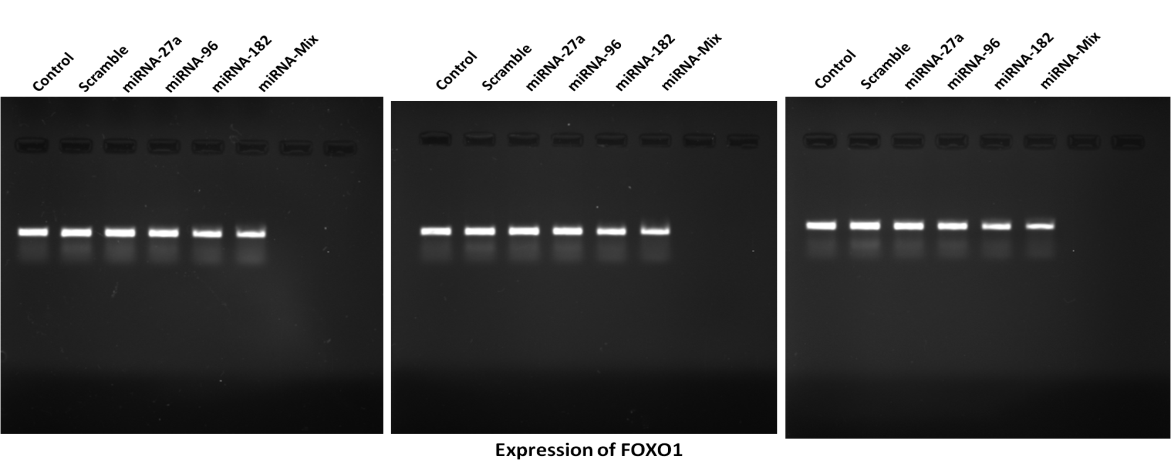

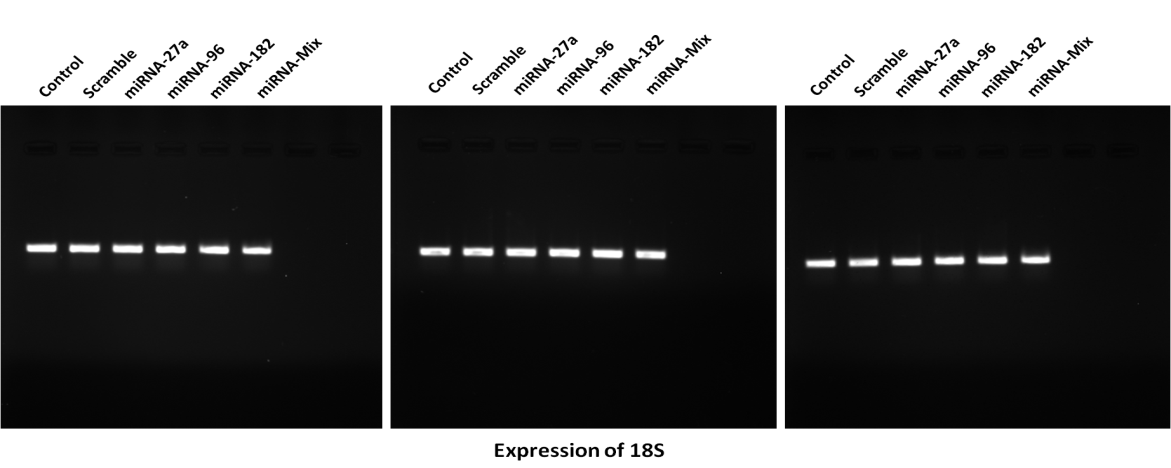
**

**Fig. 3a Gene expression in response to transfection of miRNAs.** RT-PCR products resolved in agarose gel showing the expression of 18S, FOXO1, p21, and p27 after transfected with miRs (a).

**
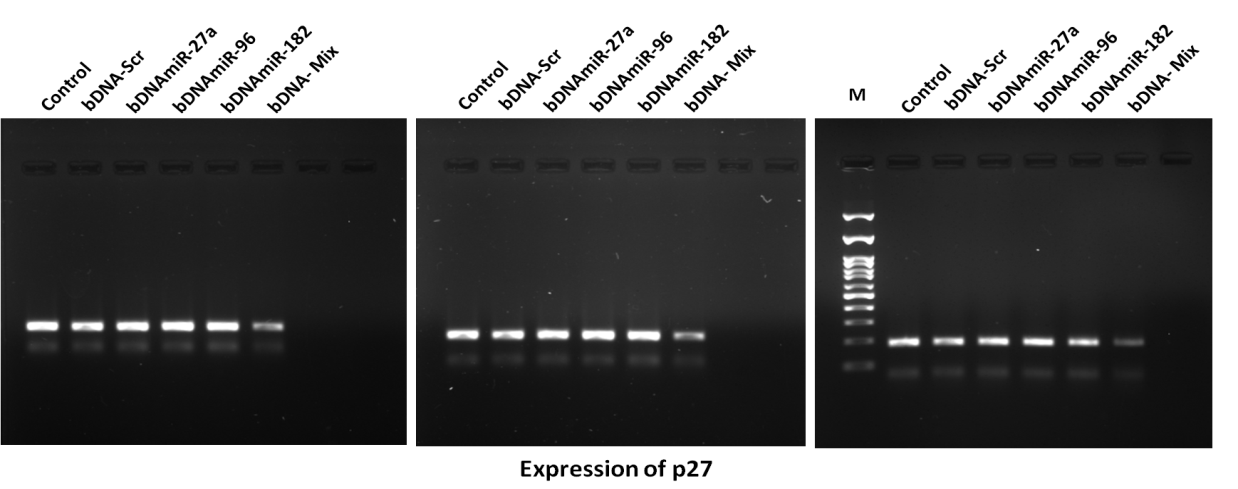

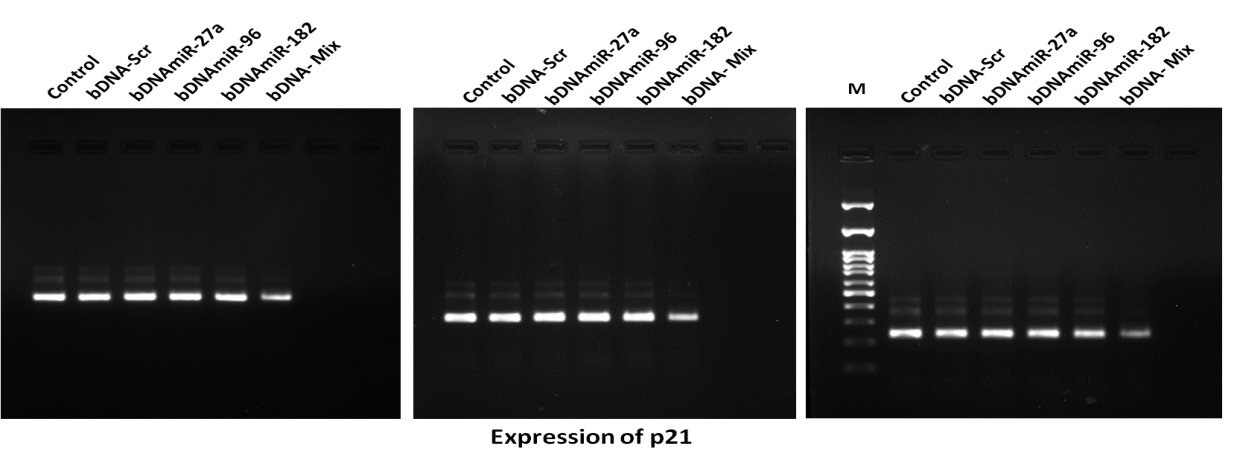

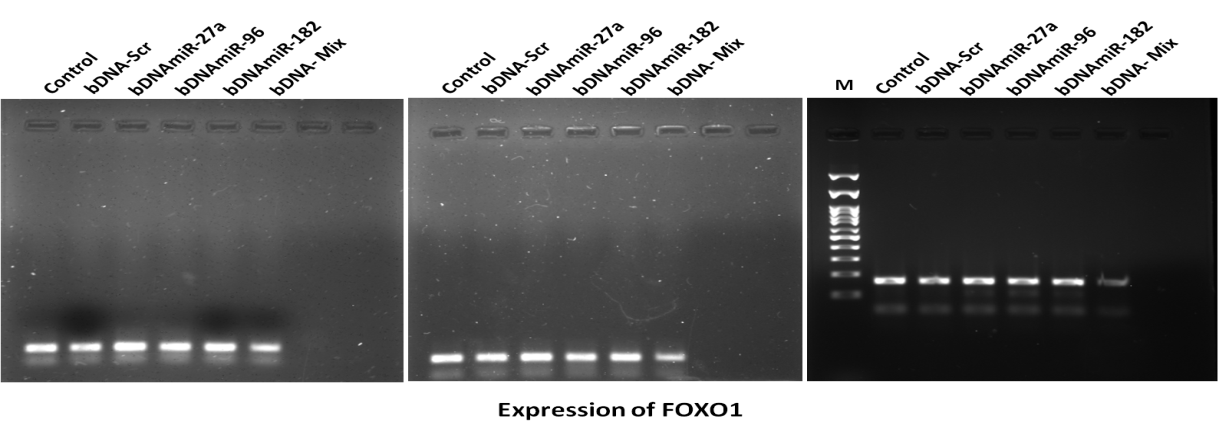

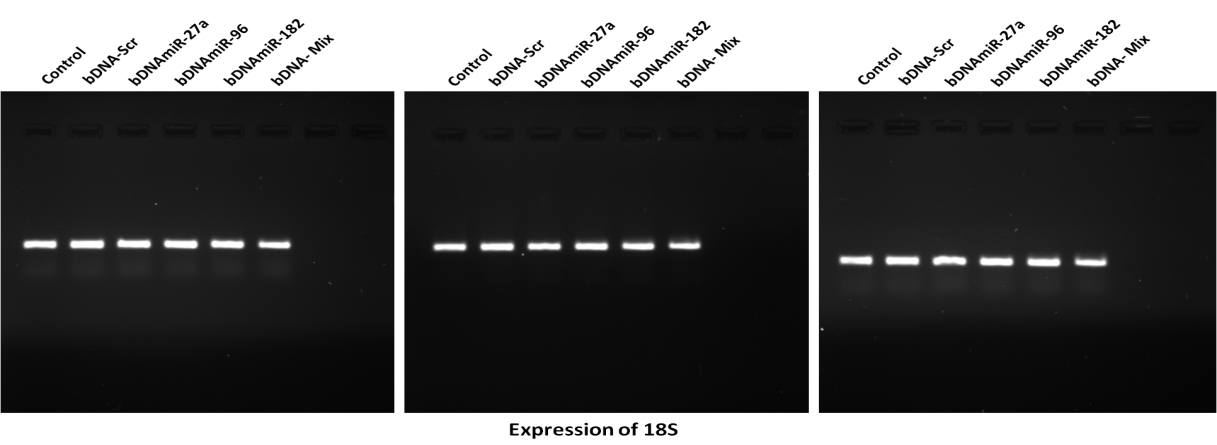
**

**Fig. 4a Transcript profile after transfection with bDNAmiR nanostructures.** Agarose gel shows the transcripts level of FOXO1, p21, p27 and 18S in response to the transfection with bDNA-miRs (a).

**Part B: Figures with full length Gel images for Supplementary Information**

**
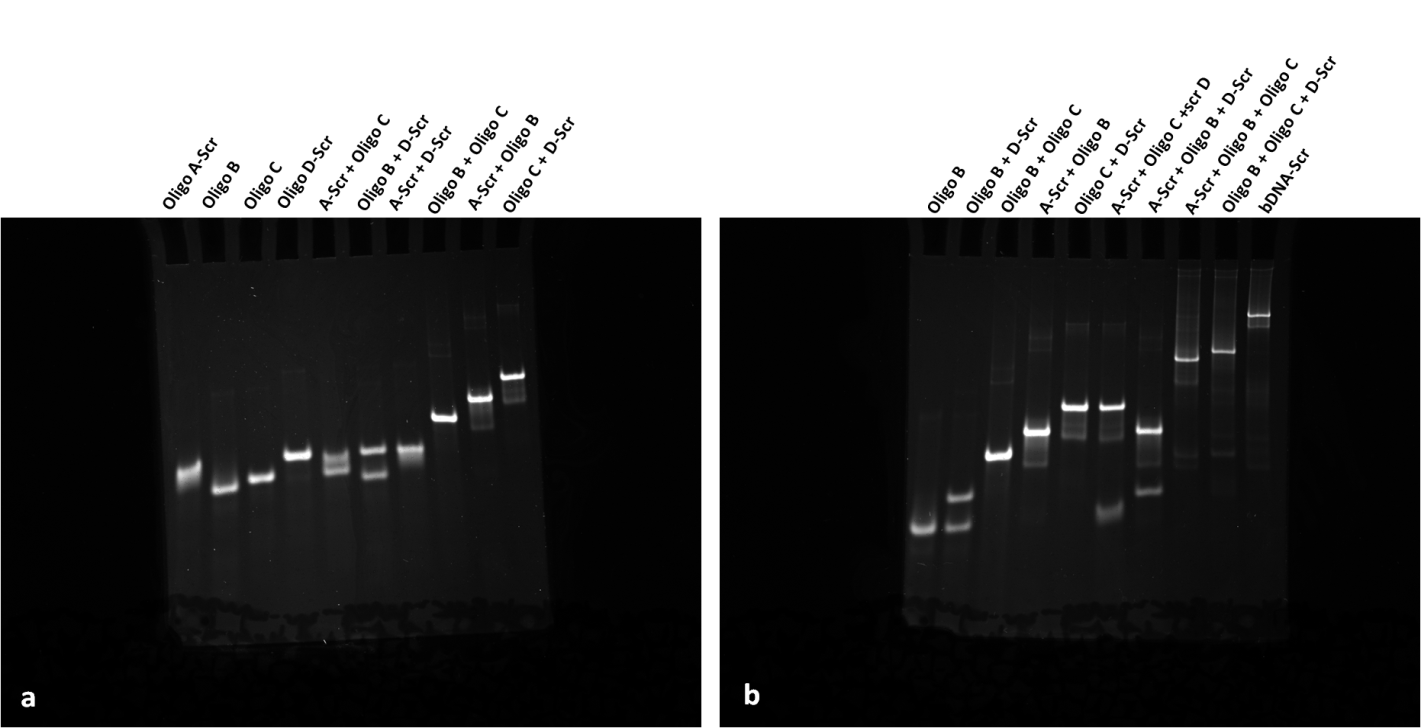

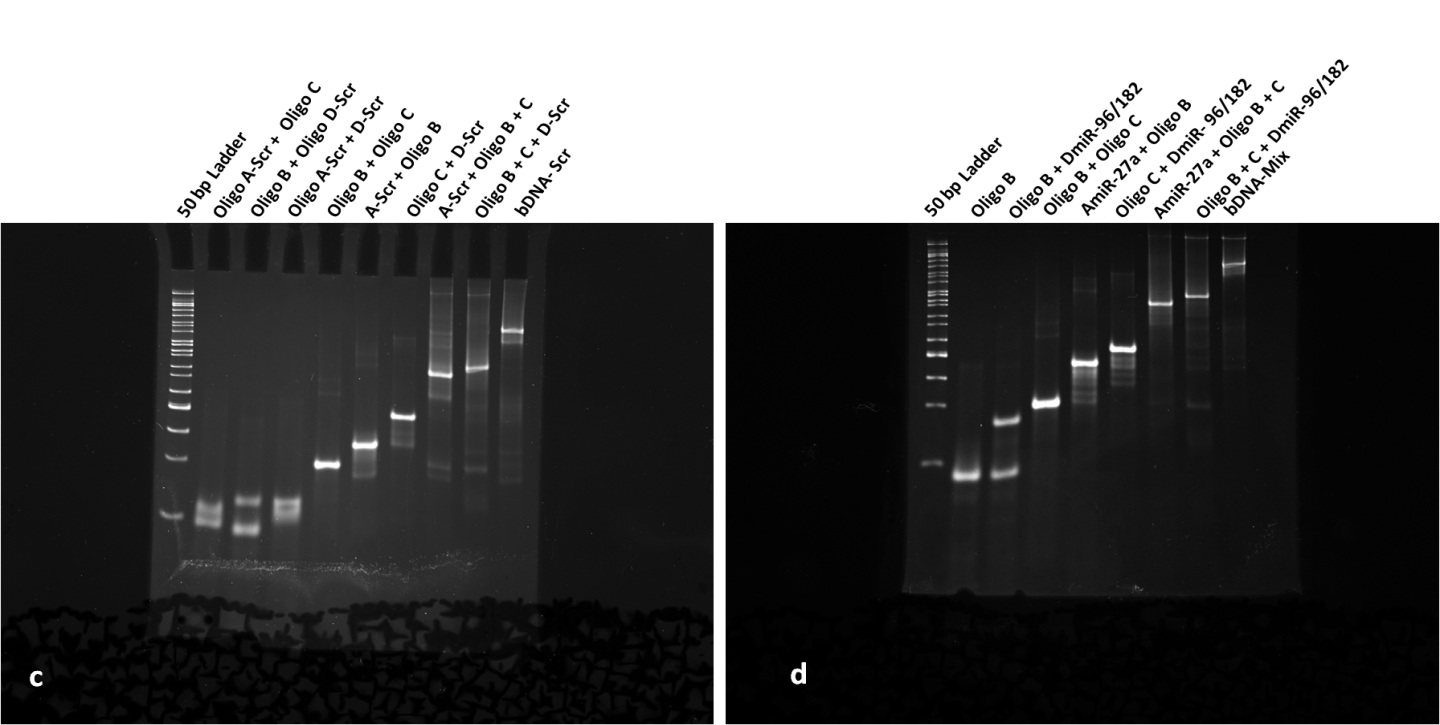
**

**Fig. S2 Characterization of self-assembled bDNA structures.** Gel image shows the intensity and integrity of single and di-oligo complexes in 10% nPAGE. Non complementary oligos shows no interaction with each other which revealed specificities of oligo designing, whereas oligos with complementary sequence results to form a desired di-oligo product (a). Characterization of single, di and tri-oligo complexes to form self-assembled bDNA-scramble in 10% nPAGE (b). Gel image showing the formation of bDNA-Scramble through the di-oligo and tri-oligo in 10% nPAGE (c). Formation of bDNA-Mix structures containing miR-27a, miR-96, miR-182 sequences in the four overhangs (d). Sample composition of each lane is mentioned on top of each lane. bDNA-Mix shows decrease electrophoretic mobility with respect to di and tri-oligo complexes. The single bands in each lane indicates precise base pairing among oligos.

**
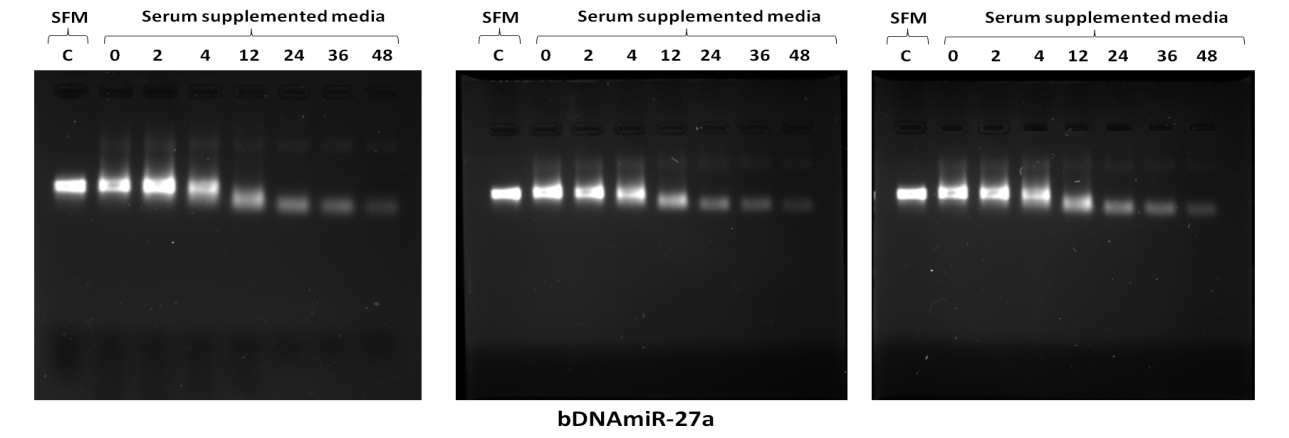

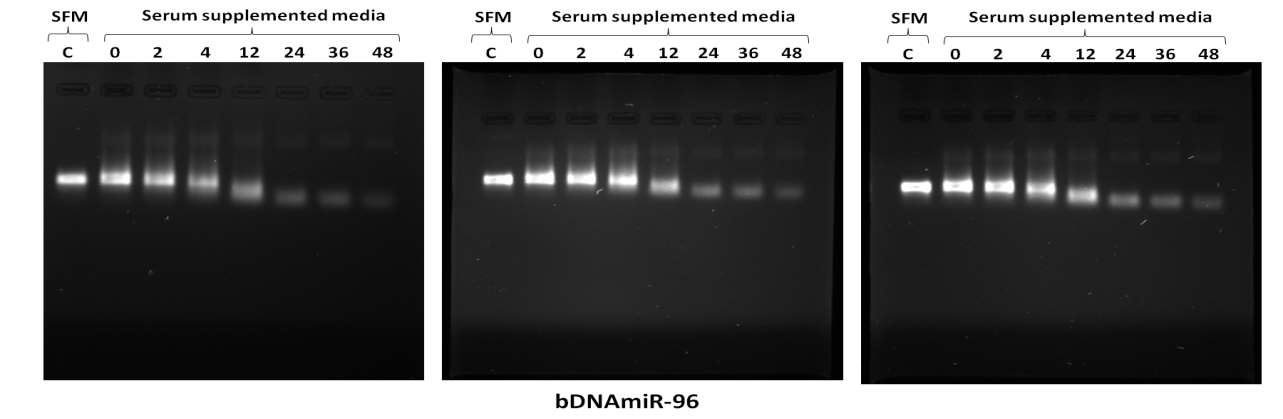

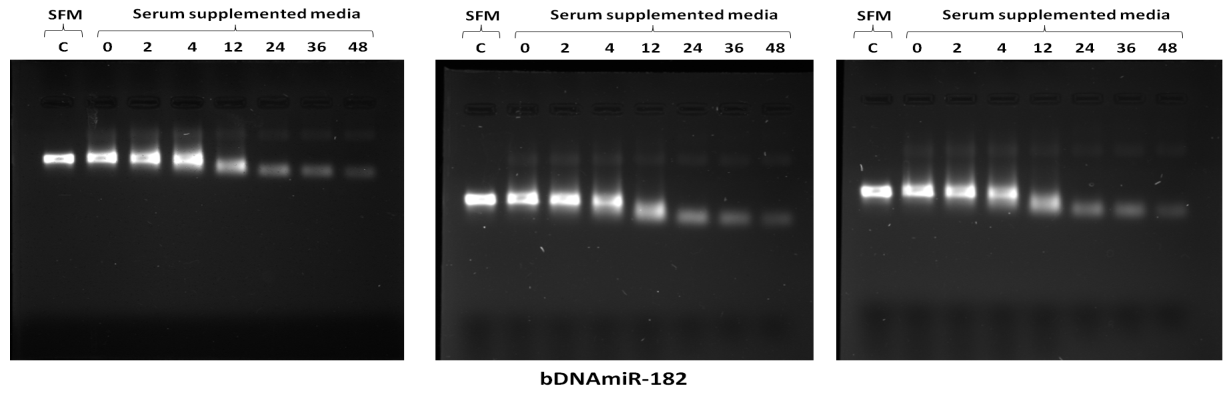

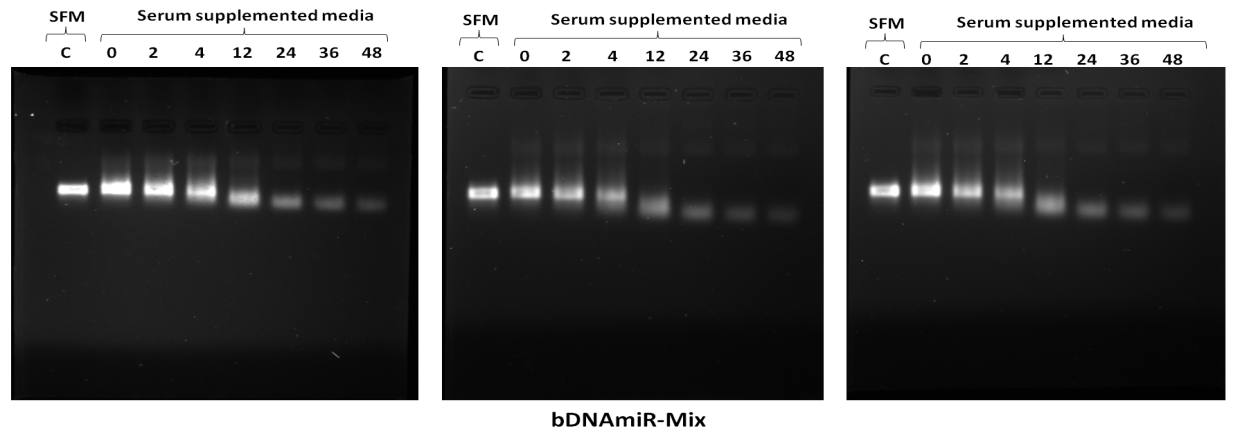
**

**Fig. S4 Serum stability assay of bDNA nanostructures.** Agarose gel image display the stability of bDNA structure after incubation at 37°C for 0 to 48 h. (SFM: Serum free media, SSM: Serum supplemented media).

**
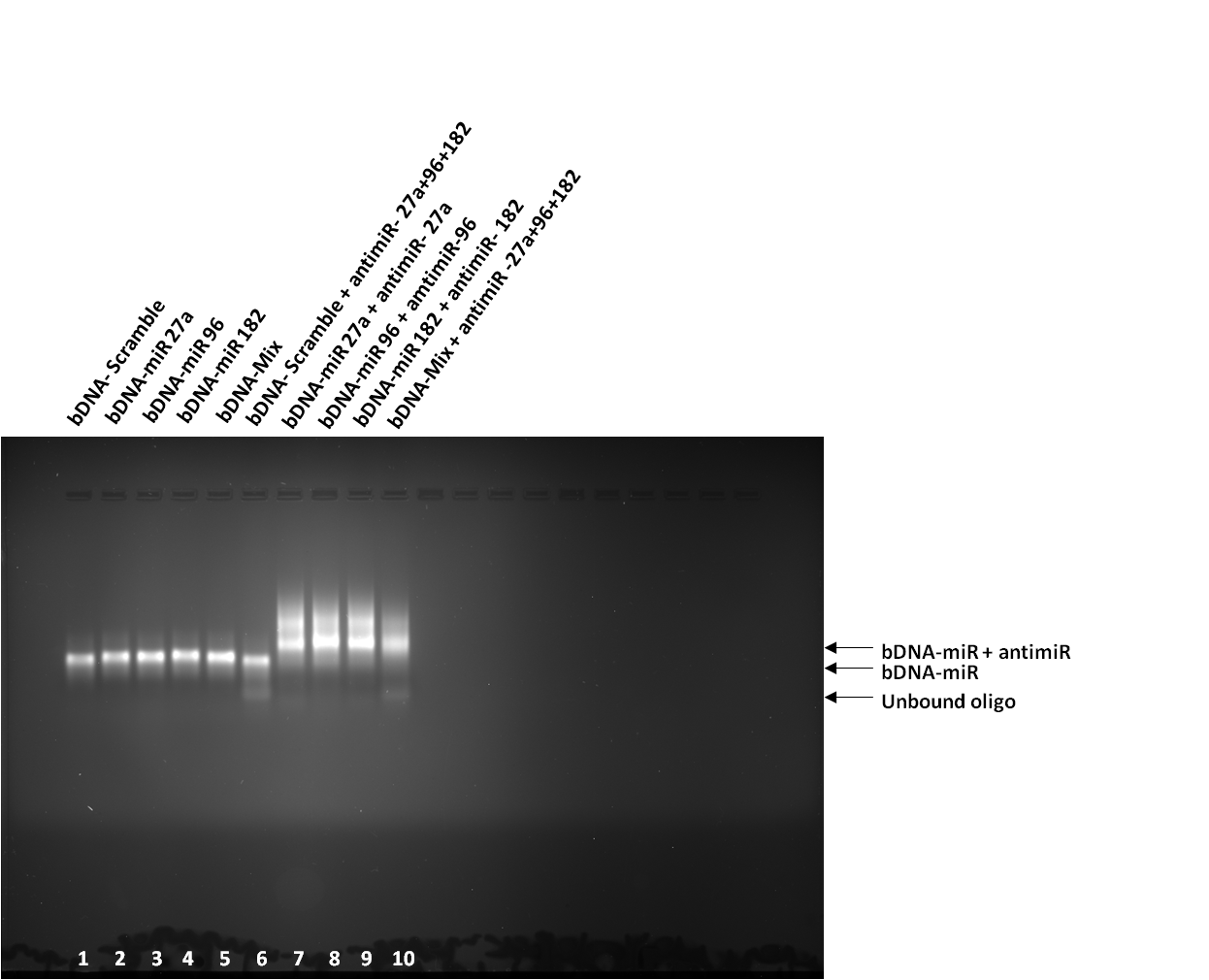
**

**Fig. S5 Gel retardation assay showing *in vitro* binding between bDNA-miR and its complementary antimiR sequences.** Gel image showing the migration of bDNA in the absence (lane 1-5), and presence of antimiRs (7-10). A clear shift of band was seen in lanes 7 to 10 due to the binding between bDNA-miR and antimiR. In bDNA-Scr (lane 6) no change in migration was observed.


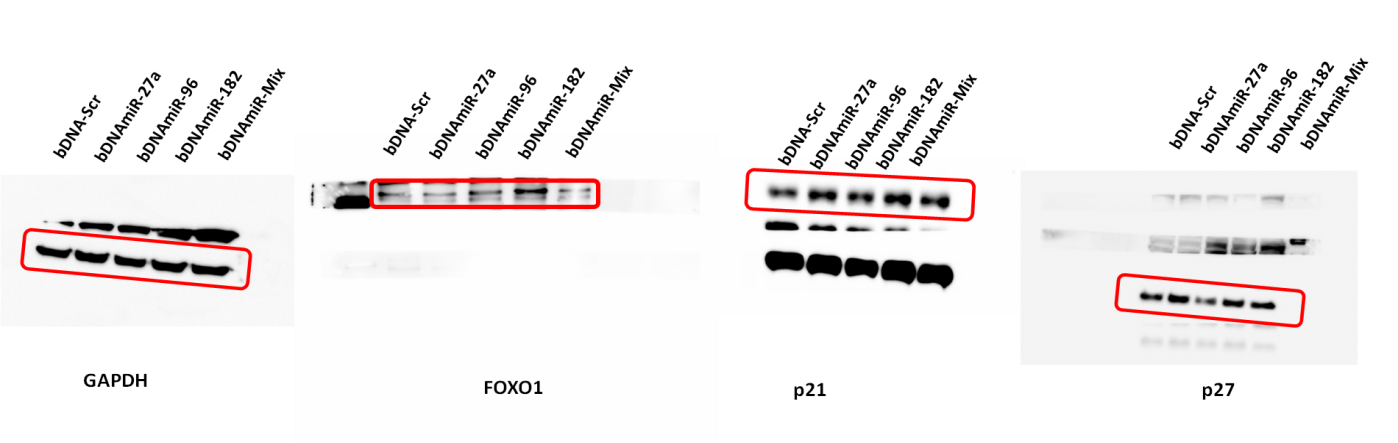


**Fig. S6** **Western blot analysis of FOXO1 Expression.** Endogenous expression of FOXO1, p21, p27 levels presented (regions highlighted in red boxes are used for the Fig. S6, SI) after transfection with bDNA-Scr and bDNA-miR (bDNAmiR-27a, bDNAmiR-96, bDNAmiR-182 and bDNA-Mix) to breast cancer MCF7 cell lines. GAPDH was used as loading control.
